# Whole genome sequencing and rare variant analysis in essential tremor families

**DOI:** 10.1371/journal.pone.0220512

**Published:** 2019-08-12

**Authors:** Zagaa Odgerel, Shilpa Sonti, Nora Hernandez, Jemin Park, Ruth Ottman, Elan D. Louis, Lorraine N. Clark

**Affiliations:** 1 Department of Pathology and Cell Biology, College of Physicians and Surgeons, Columbia University, New York, NY, United States of America; 2 Department of Neurology, Yale School of Medicine, Yale University, New Haven, CT, United States of America; 3 G.H Sergievsky Center, Columbia University, New York, NY, United States of America; 4 Department of Neurology, College of Physicians and Surgeons, Columbia University New York, NY, United States of America; 5 Department of Epidemiology, Mailman School of Public Health, Columbia University, NY, United States of America; 6 Division of Epidemiology, New York State Psychiatric Institute, New York, NY, United States of America; 7 Department of Chronic Disease Epidemiology, Yale School of Public Health, New Haven, CT, United States of America; 8 Taub Institute for Research on Alzheimer’s Disease and the Aging Brain, College of Physicians and Surgeons, Columbia University, New York, NY, United States of America; German Cancer Research Center (DKFZ), GERMANY

## Abstract

Essential tremor (ET) is one of the most common movement disorders. The etiology of ET remains largely unexplained. Whole genome sequencing (WGS) is likely to be of value in understanding a large proportion of ET with Mendelian and complex disease inheritance patterns. In ET families with Mendelian inheritance patterns, WGS may lead to gene identification where WES analysis failed to identify the causative single nucleotide variant (SNV) or indel due to incomplete coverage of the entire coding region of the genome, in addition to accurate detection of larger structural variants (SVs) and copy number variants (CNVs). Alternatively, in ET families with complex disease inheritance patterns with gene x gene and gene x environment interactions enrichment of functional rare coding and non-coding variants may explain the heritability of ET. We performed WGS in eight ET families (n = 40 individuals) enrolled in the Family Study of Essential Tremor. The analysis included filtering WGS data based on allele frequency in population databases, rare SNV and indel classification and association testing using the Mixed-Model Kernel Based Adaptive Cluster (MM-KBAC) test. A separate analysis of rare SV and CNVs segregating within ET families was also performed. Prioritization of candidate genes identified within families was performed using phenolyzer. WGS analysis identified candidate genes for ET in 5/8 (62.5%) of the families analyzed. WES analysis in a subset of these families in our previously published study failed to identify candidate genes. In one family, we identified a deleterious and damaging variant (c.1367G>A, p.(Arg456Gln)) in the candidate gene, *CACNA1G*, which encodes the pore forming subunit of T-type Ca(2+) channels, Ca_V_3.1, and is expressed in various motor pathways and has been previously implicated in neuronal autorhythmicity and ET. Other candidate genes identified include *SLIT3* which encodes an axon guidance molecule and in three families, phenolyzer prioritized genes that are associated with hereditary neuropathies (family A, *KARS*, family B, *KIF5A* and family F, *NTRK1*). Functional studies of *CACNA1G* and *SLIT3* suggest a role for these genes in ET disease pathogenesis.

## Introduction

Essential tremor (ET) is one of the most common neurological disorders. In most studies the prevalence of ET is markedly higher than that of Parkinson’s disease (PD). The prevalence of ET is estimated to be 2.2% and as much as 4.6% in cases aged >65 years [1]. Similarly, the age specific incidence is reported to increase after the age of 49 years and reaches a maximum (84 per 100 000) in the ninth decade [2]. While the majority of studies do not show a gender difference for ET, a minority of studies show a statistically significant gender difference with a higher prevalence among men than women (crude male prevalence: crude female prevalence = 1.08:1) [1]. The defining clinical feature of ET is a kinetic tremor at 4–12 Hz. This tremor occurs in the arms and hands; it may also eventually spread to involve several cranial regions (e.g., the neck, voice, and jaw). Both genetic and environmental (i.e., toxic) factors are likely to contribute to the etiology of ET. The high heritability and aggregation of ET in families suggests a Mendelian pattern of inheritance [2–5]. Family studies indicate that on the order of 30–70% of ET patients have a family history with the vast majority (>80%) of young-onset (<40 years old) cases reporting >1 affected first-degree relative [6].

Four published genome wide linkage scans have been performed all in North American or Icelandic ET families [7–9]. These studies led to the identification of genetic loci harboring ET genes on chromosomes 3q13 (ETM1 OMIM:190300) [7], 2p22-p25 (ETM2 OMIM:602134) [8], 6p23 (ETM3 OMIM: 611456) [9], and 5q35 [10]. Recently, several studies have used a whole exome sequencing (WES) approach to identify candidate genes in ET families [11–16]. Collectively, these studies suggest that ET is genetically heterogeneous.

With the limited nature of this progress, the genetic etiology of ET still remains largely unexplained. Whole genome sequencing (WGS) is likely to be of value in furthering our understanding of a large proportion of ET where WES analysis has failed to identify the causative variant [17]. WGS which forgoes capturing is less sensitive to GC content and is more likely than WES to provide complete coverage of the entire coding region of the genome [18].

Here we report analysis of eight early-onset ET families (n = 40 individuals) enrolled in the family study of Essential Tremor (FASET) at Columbia University. The analysis included filtering on WGS data based on allele frequency in population databases, rare SNV and indel classification and association using the Mixed-Model Kernel Based Adaptive Cluster (MM-KBAC) test [19, 20]. A separate analysis of rare SVs and CNVs segregating within families, prioritization of candidate genes identified within families using phenolyzer and functional studies of two candidate genes was also performed.

## Materials and methods

### Study participants and clinical diagnosis

**Fig 1:**
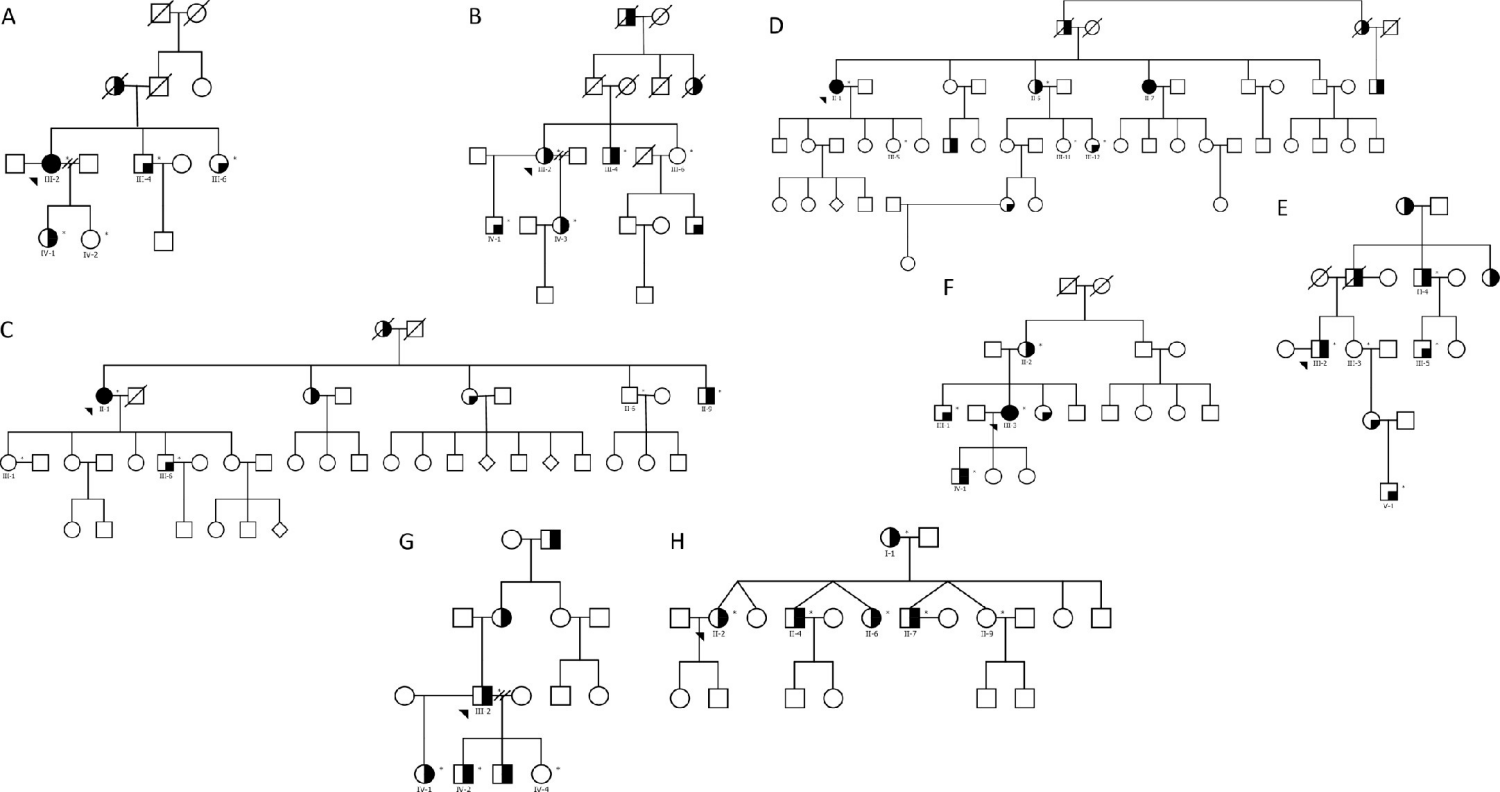
Pedigrees of eight ET families that were whole genome sequenced. Pedigrees for families (A-H) that were whole genome sequenced are shown. The generation in each pedigree is shown by roman numerals. The proband is indicated by an arrowhead. A ‘*’ symbol indicates subjects that were whole genome sequenced. Below each subject with DNA avaliable for genetic analysis the subject ID is indicated. Symbol shading is as follows: definite ET, symbols completely black; probable ET, symbols half vertical black fill; possible ET, symbols with a quadrant in black; and unaffected clear symbol. To protect the identity of participants in families the gender and birth order were changed in order to disguise their identities.

Study subjects and relatives were enrolled in a family study of ET at Columbia University NY, USA. The study was approved by the Institutional Review Board at Columbia University and written informed consent was obtained from all participants. Details of the study, criteria for enrollment, and diagnosis of ET has been described previously [15]. We selected a total of 8 families for WGS (n = 40 individuals), which included affected and unaffected first-degree relatives. A subset of the families (Families A, B and F) have been previously described in a WES study [15]. All affected individuals included in the study received a diagnosis of definite, probable or possible ET. Possible and probable ET family members were considered affected. The criteria we used, namely, the Washington Heights Inwood Genetic Study of ET (WHIGET) criteria are very strict [21]. All ET diagnoses (possible, probable and definite) required, at a minimum, moderate or greater amplitude kinetic tremor on at least three tasks, and an absence of other etiologies. As such, these criteria for all three categories of ET (i.e., possible, probable and definite) are even more stringent than those for definite ET that were outlined in the original Consensus Statement on Tremor of the Movement Disorders Society (published in 1998) [22] and the revised Consensus Criteria (published in 2017) [23]. The clinical characteristics of study participants are summarized in Table 1 and pedigrees of the families are shown in Fig 1.

**Table 1:** Clinical characteristics of affected ET individuals and unaffected family members that were whole genome sequenced in eight families.

Clinical characteristic	ET patients n = 31	Unaffected n = 9	Total n = 40
Male n (%)	12 (39)	3 (33)	15 (38)
Age at tremor onset mean years (SD)	27.83 (19.30)	NA	NA
Age at interview mean years, SD	58 (18.08)	56.63 (13.65)	57.72 (17.11)
Duration of tremor mean years, SD	30.47 (18.98)	NA	NA
Total tremor score mean SD	17.76±7.80 (39)	NA	NA
Head tremor on examination n (%)	12 (39)	NA	NA
Chin tremor on examination n (%)	6 (19)	NA	NA
Head tremor presence in head and chin n (%)	4 (13)	NA	NA

### Whole genome sequencing and quality control

Genomic DNA was isolated from peripheral blood cells using standard methods. Whole genome sequencing was performed on the genomic DNA of 4–5 individuals including affected and unaffected (definite, probable or possible ET diagnosis) individuals from each of eight families. The pedigrees of eight families are shown in Fig 1. Libraries were prepared using the TruSeq DNA PCR-free kit (Illumina San Diego CA USA). Paired-end sequencing (2x150 bp) was performed at >30x coverage per sample. Resulting libraries were sequenced on Illumina HiSeq TENx (Illuminia San Diego CA). Sequence alignment to the UCSC hg19 reference genome was performed using the Burrows-Wheeler Aligner algorithm [24] and variant calling was performed using the Genome Analysis Toolkit (GATK; Broad Institute Cambridge MA USA) [25]. Duplicate reads were removed using Picard (http://broadinstitute.github.io/picard/). Local realignment and quality recalibration was performed via GATK. Quality control checks for samples were performed according to GATK best practices.

Previously, we performed WES [15] on a subset of the families (Families A, B and F) included in the current WGS analysis. For these families, WES did not identify the candidate genes identified in the current WGS study. In S1 Table and S2 Table, we summarize the overlap of variants identified in families in both WGS and WES datasets (S1 Table) and the reason variants were not called or prioritized in the WES dataset (S2 Table).

### Variant filtering based on allele frequency in population databases

**Fig 2:**
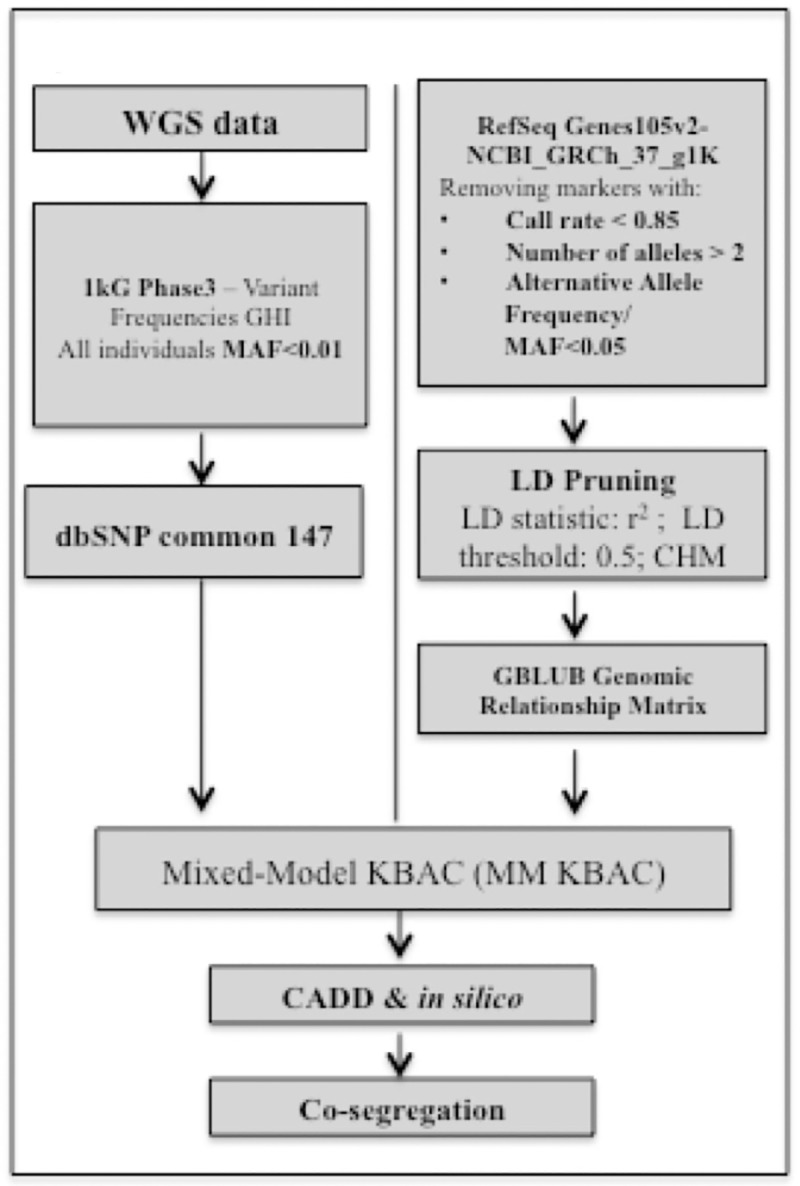
Analysis workflow for analysis using MM-KBAC. The analysis workflow for WGS data is shown with population database filtering, analysis methods and annotation.

We filtered and removed variants with MAF≥0.01 in all individuals in 1000 Genomes Phase 3 or the NCBI dbSNP common 147 database, resulting in a total of 3,777,271 rare variants across all samples (Fig 2).

#### Classification of rare variants based on variant type

Annotation of variants was performed based on reference sequence GRCh37 and RefSeq Gene transcripts of NBCI Homo sapiens Annotation Release 105 that was implemented in the Golden Helix SNP & Variation Suite (SVS) ver.8.2 (Golden Helix MT). Rare variants were classified into five groups, based on localization to a gene region and predicted effect on transcript and protein: 1) 5’-UTR and 3’-UTR (n = 26,872 variants in 8,299 genes), 2) nonsynonymous (n = 11,272 variants in 4,877 genes), 3) loss-of-function (LoF) (n = 1,365 variants in 711 genes), 4) synonymous (n = 5,854 variants in 3,164 genes), and 5) intronic (n = 1,174,082 variants in 16,486 genes). LoF variants were defined as follows: nonsense variants that introduce stop gain/loss of codons, variants that disrupt splice sites including canonical splice donor and acceptor sites and frameshift variants that disrupt a transcript’s open reading frame.

#### Annotation of variants

Rare variants were assessed using several *in silico* tools including the Combined Annotation Dependent Depletion (CADD) tool [26] implemented in the Golden Helix SNP & Variation Suite (SVS) ver.8.6.0 (Golden Helix MT) (Fig 2). CADD measures deleteriousness of variants (coding and non-coding intronic) that is a property strongly correlated with molecular functionality and pathogenicity [27]. Variants were filtered based on a phred-scaled CADD score and variants with a phred-scaled CADD score>10, corresponding to the top 10% of deleterious substitutions relative to all possible variants in the human reference genome [26] were retained for further analyses. We also assessed deleteriousness of variants using several *in silico* tools including SIFT [28], PolyPhen2 [29], LRT [30], Mutation Taster [31], FATHMM [32], PROVEAN [33], MetaSVM and MetaLR [34] as implemented in the Golden Helix SNP & Variation Suite (SVS) ver.8.6.0 (Golden Helix MT). Only variants with a phred-scaled CADD score>10 and/or predicted to be deleterious or damaging by ≥1 *in silico* prediction tool were retained for further analysis.

#### Synonymous variants in splicing regulatory regions

To determine whether synonymous variants identified in our analyses are enriched in splicing enhancer regions and splicing silencer regions we used http://genes.mit.edu/burgelab/rescue-ese/ and http://genes.mit.edu/fas-ess/ online tools, respectively [35, 36].

#### Non-coding intronic variants in DNase I hypersensitivity and transcription factor binding sites

We performed further evaluation of non-coding intronic variants by assessing whether these variants are enriched in *DNase* I hypersensitive sites that represent open chromatin regions accessible to transcription factors. We downloaded the wgEncodeRegDnaseClusteredV3 table from the DNAse Clusters track which contains DNaseI Hypersensitive Sites in 125 cell types in ENCODE (http://genome.ucsc.edu/cgi-bin/hgTables) [37].

#### Residual variation intolerance score (RVIS)

We assessed the candidate genes identified in this study to determine whether they are intolerant to variants by applying the residual variation intolerance score (RVIS) [38].

### MM-KBAC analysis

We performed a rare variant classification and association analysis using the regression and permutation based Mixed-Model Kernel-Based Adaptive Cluster method (MM-KBAC) [19], and the within gene interaction model to analyze rare functional variants, as implemented in SVS ver.8.6.0 (Golden Helix MT) (Fig 2). KBAC catalogs rare variant data within a gene region/transcript (genome-wide) into multi-marker genotypes and determines their association with the phenotype, weighing each multi-marker genotype by how often that genotype was expected to occur according to control and case data and the null hypothesis that there is no association between the genotype and the case/control status. Thus, genotypes with high sample risks are given higher weights that potentially separate causal from non-causal genotypes. The logistic mixed model approach for KBAC to adjust for family structure and relatedness was used and has been described previously [20]. Possible and probable ET family members were considered affected. The control population used included unaffected family members. A *p* value was assessed by an adaptive permutation procedure in association tests [19]. The test applied 10 000 permutations and an adaptive permutation threshold of alpha 0.01 and used the earliest start position and the last stop position of all transcripts to define a gene based on the RefSeq Gene transcripts 105v2 NCBI. By default, variants flanking (proximal and distal) the gene region up to a distance of 1000 bp were included in the analysis. We selected genes with a *p* value<0.05 for further analysis.

The analysis was performed separately for variants classified by variant type in the dataset. When MM-KBAC analysis was performed separately for variants based on variant type (nonsynonymous, LoF, 5’UTR and 3’UTR, synonymous and intronic) the total number of genes with *p* value <0.05 was 163.

### Co-segregation of variants with ET within families

Variants identified from the MM-KBAC analysis, that were annotated with a phred scaled score>10 by CADD (coding and non-coding intronic variants) and/or predicted by *in silico* prediction tools to be deleterious or damaging (coding variants) were assessed for co-segregation with ET within families (Fig 2). The criteria that we used to define co-segregation is as follows: 1) the annotated variant was present in all affected ET individuals and 2) absent from unaffected individuals within a family.

Sanger sequencing was used to validate and confirm single nucleotide variants and small indel variants within a family and to genotype family members with available DNA that did not have WGS data.

Genes harboring variants that were annotated with a phred scaled score>10 by CADD (coding and non-coding intronic variants) and/or predicted by *in silico* prediction tools to be deleterious or damaging (coding variants) and that co-segregated with ET within single family were prioritized for phenolyzer.

### Prioritization of candidate genes using phenolyzer

Phenolyzer is a computational tool that uses prior information to implicate genes involved in diseases [39]. Phenolyzer exhibits superior performance over competing methods for prioritizing Mendelian and complex disease genes based on disease or phenotype terms entered as free text. The most disease relevant genes, considering all reported gene-disease relationships, are shown as seed genes. Predicted genes are input (seed) genes that are expanded to include related genes on the basis of several gene-gene relationships (e.g. protein-protein interactions, biological pathway, gene family or transcriptional regulation). The following disease/phenotype terms were used: Tremor, Essential Tremor, Parkinson’s disease, Channelopathy, Epilepsy, neurological, neurodegenerative, Spinocerebellar ataxia, Fragile X Associated Tremor Ataxia Syndrome, brain, cerebellar diseases. For each family, candidate genes with prioritized variants were uploaded as input for phenolyzer analysis. The gene disease score and gene prediction score system is described in Yang et al., 2015 [39]. Phenolyzer generates raw and normalized scores for seed and predicted genes [39].

### SV and CNV detection algorithms

#### CNV calling

To identify germline genic SVs and CNVs from short read WGS data we adhered to the recommended best practices workflow described by Trost et al., 2018 [40].

The SV and CNV-detection algorithms used were Canvas version 1.19.1 and Genome STRucture in Populations (Genome STRiP) version 2.0. The Canvas algorithm was run using the Germline-WGS module. B-allele frequencies were from the dbsnp.vcf provided with Canvas software. Custom parameters were used for the Canvas Bin and Canvas partition algorithms. The Binary Bin algorithm and the Circular Binary Segmentation Partitioning algorithm were used. Low confidence calls with low supporting bins (BC<8) were filtered from the VCF before analysis. For the GenomeSTRiP CNV command module a minimum refined size of 500bp was used as a cutoff. For the GenomeSTRiP SV command module, SVs were called in two size ranges as recommended by best practices (100–100,000bp and 100,000–10,000,000bp). The overlap and intersect of SVs and CNVs from the two algorithms was determined per sample.

To identify rare genic SVs and CNVs segregating with ET in each family a family based analysis was performed using Clinical Reporter in Opal (Fabric genomics, Inc.). The criteria that we used to define co-segregation is as follows: 1) the annotated CNV was present in all affected ET individuals and 2) absent from unaffected individuals within a family.

#### Selecting rare or novel CNVs

Common variants were removed following comparison with the Database of Genomic Variants (DGV) and 1000 Genomes CNV calls, and using a criterion of 50% or more reciprocal overlap with population CNVs with 1% or higher frequency. BEDTools [41] was used to identify called genic CNVs that overlapped with variants in databases.

#### Reducing false calls

To minimize false calls, rare genic CNV calls (consensus of Genome STRiP v2.0 and Canvas v1.38.0) from each affected individual was used to query calls in affected and unaffected family members for the same or similar breakpoints. If the same CNV (consensus of the two tools) was present in affected individuals and absent from unaffected individuals it was included in the final list.

#### Annotation of CNV calls

Annotation of CNV calls was performed using Opal (Fabric genomics, Inc.) and included variant effect predictor (VEP) which determines the effect of the variant on genes, transcripts and protein sequences as well as regulatory regions [42], population databases including the Database of Genomic Variants (DGV) and dbVar (National Center for biotechnology information), and literature evidence (OMIM, ClinVar, COSMIC, etc).

#### *De novo* CNV calling

*De novo* CNV calling was not performed as sequence data was not available from both parents. Trio families were not included in the analysis.

### Functional studies of *SLIT3* and *CACNA1G*

#### *Slit Drosophila* lines

Generation of drosophila *slit* lines was performed by GenetiVision Corporation (Houston, TX). To determine the pathogenicity of the *SLIT3* variant (c.3505G>C (NM_001271946.1), p.(Val1169Leu)(*Drosophila* slit p.Val1187Leu; NP_001261017.1) that we identified in family D, *Drosophila slit* lines were created via two steps of CRISPR-Cas9 mediated homology directed repair (HDR) events. In the first step, two crispr/cas9 targets were designed to delete a 183 bp fragment containing the V1187. Two guide RNAs (TGCTTCCAACCGAACGCTCA & AATGGAATTCTCATGTACGA) were cloned into pCFD3 vector (http://www.flyrnai.org/tools/grna_tracker/web/files/Cloning-with-pCFD3.pdf) and a donor DNA was created with our GFP cassette flanked by two 1 kb SLIT sequences beyond cleavage sites. Upon co-injection of both DNA constructs, two gRNAs will be expressed to direct the double strand break (DSB) by cas9 (endogenously expressed in the injection stock BL#54591). After DSB, the GFP cassette was inserted into SLIT genome via donor DNA mediated recombination. In the second step, based on the same principle, GFP KI cassette was substituted by a 420 bp DNA fragment containing V1187L point mutation using a new set of gRNAs (CCGCTGTCCAGACGACTATA & CGATGGAAAGTACCATGCCG) and new donor DNA. A molecular screen to confirm the mutation involving 20 individual mating crosses followed by genomic DNA isolation of the founder, PCR and sequencing was used to confirm the presence of the mutation. We independently confirmed the presence of the mutation by Sanger sequencing. Briefly, flies (n = 2) were homogenized in AL buffer from the DNeasy Blood and Tissue kit (Qiagen, Germany) and processed for genomic DNA according to the manufacturers instructions. PCR amplification of the region containing the point mutation was amplified and cloned using the Original TA cloning kit (Invitrogen, CA). DNA isolated from randomly selected clones were Sanger sequenced. Sequencing chromatograms showing the point mutation are provided as supplementary data (S1 Fig).

#### Negative geotaxis climbing assay

The loss of climbing response was used to monitor aging related locomotor changes in Drosophila [43, 44]. The climbing assay was performed as previously described [43, 44]. We assessed 10 flies per vial for each *slit* mutant line and control line. 5 trials were conducted for each vial. The average climbing rate was determined by measuring the first fly to climb 17.5cm. Climbing response was assessed at the following time points: Day 7, 14, 21, 28, 35 and 42.

#### Lifespan assay

Lifespan assays were performed as described previously [45]. Briefly, 100 virgin male flies per line were sex-segregated within 4h of eclosion and maintained in small laboratory vials (n = 20 per vial) containing fresh food in a low-temperature incubator at 25°C and 40% humidity on a 12/12h dark/light cycle. The flies were then transferred to fresh food vials every 2–3 days and mortality recorded.

### Ca_V_3.1 electrophysiology

#### Transfection and cell culture procedures

To determine the functional effects of *CACNA1G* variants identified in ET families on electrophysiology studies by whole cell patch clamp recordings was performed in HEK293 cells expressing the Ca_V_3.1 mutant or wild type channels. The variants rs200317339 (c.2271G>A; p.Gly627Arg), rs116920450 (c.1759G>A; p.Arg456Glu) and rs150972562 (c.4096G>A; p.Arg1235Gln) were introduced into human wild-type *CACNA1G* (isoform 1;BC110995.1, NM_198382.2) by site directed mutagenesis (KIT details). HEK293 cells were cultured in 150mm dishes until 75% confluence. For the transfection reaction cells were harvested, counted and resuspended in MaxCyte buffer to reach 50 million cells per ml. DNA of wild type or mutant Cav3.1 channels were added to the cell suspension to a final concentration of 100μg per ml. Electroporation was performed using the MaxCyte machine. Following the reaction cells were transferred into a 48-well plate to recover in electroporation buffer containing DNase for 20 minutes and in recovery media for 60 min both steps at 37°C. After recovery cells were counted and plated in 35mm dishes for manual patch clamp studies. Electrophysiological studies were performed 24–72 hours post plating.

#### Electrophysiology

Experiments were performed at room temperature or near-physiological temperature. Four voltage protocols (1) activation kinetics, 2) deactivation and inactivation kinetics, 3) steady state inactivation and 4) voltage dependence of recovery from inactivation) were applied to at least four cells expressing the wild type channel (n = 4) and four cells expressing the mutant channel (n = 4). For the voltage protocol designed to investigate activation kinetics currents are activated from a -100 mV holding potential by 60 ms step pulses in 10mV increments up to +70mV. Investigation of deactivation and inactivation kinetics was performed by maximal activation of currents by 2 ms step pulses to +60mV (P1) followed by 38 ms pulses (P2) from +60mV to -120mV in 10mV steps. The current activated at P3 gauges the amount of inactivation developed during P2. To investigate steady state inactivation, the ratio test (P2) to control (P1) currents activated by 5ms step pulses to -20mV was used as an indicator of the fraction of channels inactivated during the 1s pulses (P2) from -120 to -40mV. In protocol 4, to investigate the voltage dependence of recovery from inactivation, inactivation was induced by a 60 ms step pulse to -20mV (P1) and gauged by the current elicited by a 10 ms pulse to -20 mV (P3) after a variable period of recovery at -120, -80 and -70 mV (P2).

#### Data analysis

Data acquisition and analyses was performed using the suite of pCLAMP (Ver. 8.2) programs (MDS-AT, Sunnyvale, CA).

#### Activation

Activation was parametrized by the voltage dependence of the peak current amplitude and the time to peak (TP) of currents activated using protocol 1. The voltage dependence of peak negative current amplitude was fitted to the following equation:

I(V)=Gmax(V−Er)∕[1+exp(−(V−Vhalf)∕k)]

Where V is the membrane potential in mV, Vhalf is the membrane potential where half of the channels are activated, k is the slope factor in mV, Gmax is the maximal conductance in nS and Er is the apparent reversal potential in mV.

The voltage dependence of the rate of channel opening parametized by TP was fitted to the following equation:

$$TP(V)=exp[-(V-V_{TP})∕k]+{TP}_{\infty }$$

Where V_TP_ is the voltage at which TP is equal to 1+TP∞ and k is the voltage sensitivity in mV.

#### Deactivation

Channel closing was assessed using currents activated with protocol 2. The voltage dependence of channel closing was evaluated fitting the time constants of tail currents between -70mV and -120mV or the minimal membrane potential where tail currents were resolved with the following equation:

$$\tau (V)=\{exp[-(V-V\tau_{deact})∕k]{\}}^{‐1}$$

Where *Vτ*_*deact*_ is the potential at which *τ* is equal to 1 and k is the voltage sensitivity in mV.

#### Inactivation

Channel inactivation will be parametrized by 1) the voltage dependence of the steady state inactivation curve from currents elicited using protocol 3, 2) the time constant of inactivation from currents recorded at positive potentials during the P2 step described in protocol 2 and 3) by the voltage dependence of the rate of recovery from inactivation measured using protocol 4.

The voltage dependence of steady state inactivation will be fitted to the equation:

$$P2∕P1=\{1+exp[(V-V_{half})∕k]{\}}^{‐1}$$

Where P2/P1 is the ratio of peak currents elicted by the test and control steps, V is the membrane potential in mV, *V*_*half*_ is the membrane potential where 50% of the channels are inactivated and k is the slope factor in mV.

The voltage dependence of the inactivation time constants will be evaluated by plotting the functional relationship between *τ* inact and V emphasizing on possible changes in the voltage -independent limiting rate.

The rate of recovery from inactivation will be evaluated fitting a single exponential function according the following equation:

$$P3/P1=A(1-exp(-t∕\tau_{rec}))$$

Where P3/P1 is the ratio of peak currents elicited by the test and control steps, A is the P3/P1 ratio after complete recovery at the corresponding potential and *τ*_*rec*_ is the characteristic time constant of recovery from inactivation at a particular voltage.

### Availability of data

All phenotype data generated from this study has been deposited in the database of Genotypes and Phenotypes (dbGaP; http://www.nlm.nih.gov/gap) of the National Center for Biotechnology Information. The study titled ‘Identification of Susceptibility Genes for Essential Tremor’ received the dbGaP Study Accession: phs000966.v2.p1. Additionally, all deidentified WGS data and related meta data underlying the findings reported in this manuscript are available at the public repository Dryad (datadryad.org) with DOI number doi:10.5061/dryad.td8d20v.

## Results

To identify candidate genes in ET we conducted WGS in 40 individuals from 8 families with multiple affected ET members (Table 1 and Fig 1). Datasets were generated based on filtering of variants on allele frequency in population databases (Fig 2). To identify and prioritize genes in the ET families we performed rare variant classification and association analysis using the Mixed-Model Kernel Based Adaptive Cluster (MM-KBAC) test [19] followed by phenolyzer [39].

### Rare variant classification and association analysis of rare variants with MAF<0.01

After QC and variant filtering, a total of 3,777,271 variants were selected for the subsequent analyses (Fig 2). By MM-KBAC analysis, we obtained 1,325 genes with *p* value<0.05 (with-in gene association) and 3,779 variants located within these genes. Of those, 783 variants were annotated with a phred scaled score>10 by CADD and 95 variants were predicted by *in silico* prediction tools to be deleterious or damaging.

We assessed the following variant types: 1) nonsynonymous, 2) LoF, 3) 5’UTR and 3’UTR, 4) synonymous and 5) intronic variants. Variants identified from the MM-KBAC analysis, that were annotated with a phred scaled score>10 by CADD and/or predicted by *in silico* prediction tools to be deleterious or damaging and that co-segregated within the ET families are shown in Table 2. A total of 168 variants located in 163 genes co-segregated with ET within families.

**Table 2:** Variants identified in families co-segregating with ET based on MM-KBAC analysis of rare variants by variant type.

Ch	Position	Ref	Alt	Gene Names	RefSeq accession or cDNA (*HGVS*)	Protein (*HGVS*)	Family
11	62283386	A	C	*AHNAK*	NM_001620.2:c.*830T>G		A
12	4735970	A	.	*AKAP3*	NM_006422.3:c.2098delT	p.(Ser700fs) (NP_006413.3)	A
11	46450229	G	A	*AMBRA1*	NC_000011.9 (NM_001267782.1*)*:c.2985+4795C>T		A
8	68128883	C	T	*ARFGEF1*	NM_006421.4:c.4628G>A	p.(Arg1543Gln) (NP_006412.2)	A
17	34325326	G	T	*CCL15*	NM_032965.4:c.238C>A	p.(Pro80Thr) (NP_116741.1)	A
8	25902635	C	T	*EBF2*	NM_022659.3:c.-260G>A		A
1	152283742	G	T	*FLG*	NM_002016.1:c.3620C>A	p.(Ser1207Tyr) (NP_002007.1)	A
4	57514946	G	A	*HOPX*	NM_001145460.1:c.*7C>T		A
16	75663435	G	A	*KARS*	NM_001130089.1:c.1513C>T	p.(Arg505Cys) (NP_001123561.1)	A
17	47302438	C	A	*PHOSPHO1*	NM_001143804.1:c.49G>T	p.(Gly17Trp) (NP_001137276.1)	A
2	131221045	C	T	*POTEI*	NM_001277406.1:c.2572G>A	p.(Gly858Arg) (NP_001264335.1)	A
8	53321917	C	T	*ST18*	NM_014682.2:c.-527G>A		A
17	20914484	C	T	*USP22*	NM_015276.1:c.1083G>A	p.(Thr361 = ) (NP_056091.1)	A
11	66053439	C	T	*YIF1A*	NC_000011.9 *(*NM_020470.2*)*:c.428-210G>A		A
11	46723055	-	TT	*ZNF408*	NM_024741.*2*:c.158_159insTT	p.(Leu54fs) (NP_079017.1)	A
8	24193085	G	A	*ADAM28*	NM_014265.4:c.1498G>A	p.(Gly500Arg) (NP_055080.2)	B
5	73207339	C	G	*ARHGEF28*	NM_001080479.2:c.4887C>G	p.(Ala1629 = ) (NP_001073948.2)	B
11	379948	C	T	*B4GALNT4*	NM_178537.4:c.2571C>T	p.(Ser857 = ) (NP_848632.2)	B
1	92445126	G	A	*BRDT*	NM_001242806.1:c.1111G>A	p.(Asp371Asn) (NP_001229735.1)	B
2	241536279	C	T	*CAPN10*	NM_023083.3:c.1663C>T	p.(Arg555Cys) (NP_075571.1)	B
9	70483186	A	G	*CBWD5*	NM_001024916.2:c.430+2T>C		B
9	130953056	-	^α^	*CIZ1*	NM_001257975:c.148_171dupTTACGCAGCAGCAGCTCCAGCAG	p.(Gln57_Gln58insLeuGlnGlnGlnGlnLeuGlnGln) (NP_001244904.1)	B
1	98205947	C	T	*DPYD*	NM_000110.3:c.321+1G>A		B
9	130272601	G	C	*FAM129B*	NM_022833.*2*:c.985C>G	p.(Pro329Ala) (NP_073744.2)	B
12	49943258	G	A	*KCNH3*	NM_012284.1:c.1503G>A	p.(Thr501 = ) (NP_036416.1)	B
12	57975211	G	A	*KIF5A*	NM_004984.2:c.2769G>A	p.(Arg923 = ) (NP_004975.2)	B
12	53008439	G	A	*KRT73*	NM_175068.2:c.743C>T	p.(Thr248Met) (NP_778238.1)	B
8	23177415	C	G	*LOXL2*	NM_002318.2:c.1453G>C	p.(Ala485Pro) (NP_002309.1)	B
5	1477557	G	A	*LPCAT1*	NM_024830.3:c.861C>T	p.(Pro287 = ) (NP_079106.3)	B
16	58537777	A	G	*NDRG4*	NM_001130487.1:c.253A>G	p.(Ile85Val) (NP_001123959.1)	B
2	131221215	T	A	*POTEI*	NM_001277406.1:c.2402A>T	p.(His801Leu) (NP_001264335.1)	B
2	113940482	C	T	*PSD4*	NM_012455.2:c.449C>T	p.(Thr150Met) (NP_036587.2)	B
19	2251466	C	T	*AMH*	NM_000479.3:c.1193C>T	p.(Pro398Leu) (NP_000470.2)	C
18	55362414	-	A	*ATP8B1*	NM_005603.4:c.929dupT	p.(Ile311fs) (NP_005594.1)	C
7	107112470	C	T	*COG5/GPR22*	NC_000007.13(NM_006348.3):c.631+55212G>A(NM_005295.2):c.304C>T		C
3	148552329	C	T	*CPB1*	NM_001871.2:c.192C>T	p.(His64 = ) (NP_001862.2)	C
2	70524477	G	C	*FAM136A*	NM_032833.2:c.361C>G	p.(Leu121Val) (NP_116211.2)	C
8	33229632	C	T	*FUT10*	NM_032664.3:c.*463G>A		C
19	35645021	C	T	*FXYD7*	NM_022006.1:c.*202C>T		C
4	6864479	C	T	*KIAA0232*	NM_014743.2:c.2370C>T	p.(Ser790 = ) (NP_055558.2)	C
7	98792785	T	A	*KPNA7*	NM_001145715.1:c.461A>T	p.(Glu154Val) (NP_001139187.1)	C
19	3786257	G	A	*MATK*	NC_000019.9 (NM_002378.3*)*:c.76-1375C>T		C
8	16012594	G	A	*MSR1*	NM_138715.2:c.877C>T	p.(Arg293Ter) (NP_619729.1)	C
15	23014502	C	T	*NIPA2*	NM_030922.6:c.223G>A	p.(Ala75Thr) (NP_112184.4)	C
3	135721907	A	G	*PPP2R3A*	NM_002718.4:c.1567A>G	p.(Met523Val) (NP_002709.2)	C
17	45992740	G	A	*SP2*	NM_003110.5:c.70G>A	p.(Ala24Thr) (NP_003101.3)	C
17	43922409	A	G	*SPPL2C*	NM_175882.2:c.137A>G	p.(Tyr46Cys) (NP_787078.2)	C
6	144508380	G	A	*STX11*	c.616G>A (NM_003764.3)	p.(Glu206Lys) (NP_003755.2)	C
7	27809333	G	A	*TAX1BP1*	NM_006024.6:c.492G>A	p.(Leu164 = ) (NP_006015.4)	C
12	101685524	C	T	*UTP20*	NM_014503.2:c.896C>T	p.(Ser299Leu) (NP_055318.2)	C
12	118533479	G	A	*VSIG10*	NM_019086.5:c.220C>T	p.(Arg74Trp) (NP_061959.2)	C
17	44950096	C	T	*WNT9B*	NM_003396.1:c.291C>T	p.(Arg97Arg) (NP_003387.1)	C
5	179105676	C	T	*CBY3*	NM_001164444.1:c.637G>A	p.(Ala213Thr) (NP_001157916.1)	D
20	56096790	G	A	*CTCFL*	NC_000020.10(NM_001269043.1):c.754+1334C>T		D
9	140611424	C	T	*EHMT1*	NM_024757.4:c.432C>T	p.(Ala144 = ) (NP_079033.4)	D
3	184290726	C	T	*EPHB3*	NM_004443.3:c.618C>T	p.(Arg206 = ) (NP_004434.2)	D
14	100118616	T	C	*HHIPL1*	NM_001127258.1:c.311T>C	p.(Leu104Pro) (NP_001120730.1)	D
17	9143279	G	A	*NTN1*	NM_004822.2:c.1809G>A	p.(Lys603 = ) (NP_004813.2)	D
8	68972914	C	T	*PREX2*	NM_024870.2:c.1239C>T	p.(Ser413 = ) (NP_079146.2)	D
6	110759925	G	-	*SLC22A16*	NM_033125.3:c.1309delC	p.(Gln437fs) (NP_149116.2)	D
5	168112742	C	G	*SLIT3*	NM_003062.3:c.3505G>C	p.(Val1169Leu)(NP_003053.1)	D
3	185211778	-	C	*TMEM41A*	NC_000003.11 *(*NM_080652.3*)*:c.574+633dupG		D
9	139820182	C	T	*TRAF2*	NM_021138.3:c.1335C>T	p.(Asp445 = ) (NP_066961.2)	D
3	180320969	G	A	*TTC14*	NM_133462.3:c.344G>A	p.(Arg115Gln) (NP_597719.1)	D
17	67039819	G	T	*ABCA9*	NM_080283.3:c.611C>A	p.(Ser204Ter) (NP_525022.2)	F
16	2578297	C	T	*AMDHD2*	c.778C>T (NM_001145815.1)	p.(Arg260Cys) (NP_001139287.1)	F
6	109200145	^β^	-	*ARMC2*	NC_000006.11(NM_032131.4):c.671+2592_671+2611delCATCCACCCAGACACCCATT		F
11	76750976	T	A	*B3GNT6*	NM_138706.4:c.381T>A	p.(Pro127 = ) (NP_619651.3)	F
17	80918994	C	T	*B3GNTL1*	NM_001009905.1:c.664G>A	p.(Val222Met) (NP_001009905.1)	F
22	30116623	G	A	*CABP7*	NC_000022.10(NM_182527.2*)*:c.109+101G>A		F
14	103404716	C	T	*CDC42BPB*	NM_006035.3:c.4860G>A	p.(Pro1620 = ) (NP_006026.3)	F
6	35765011	G	A	*CLPS*	NM_001832.3:c.55C>T	p.(Pro19Ser) (NP_001823.1)	F
19	15770059	C	A	*CYP4F3*	NM_000896.2:c.1427C>A	p.(Ala476Glu) (NP_000887.2)	F
1	100679506	A	-	*DBT*	NC_000001.10(NM_001918.3*)*:c.939+867delT		F
17	7722271	G	A	*DNAH2*	NM_020877.2:c.10705G>A	p.(Asp3569Asn) (NP_065928.2)	F
11	75167849	AT	-	*GDPD5*	NM_030792.6:c.327_328delAT	p.(Cys110fs) (NP_110419.5)	F
19	14593508	G	A	*GIPC1*	NM_005716.3:c.281C>T	p.(Thr94Ile) (NP_005707.1)	F
6	42146612	A	G	*GUCA1A*	NM_000409.3:c.424A>G	p.(Lys142Glu) (NP_000400.2)	F
1	156814612	T	C	*INSRR/NTRK1*	NC_000001.10(NM_014215.2*)*:c.2461A>G(NM_001007792.1*)*:c.122+2627T>C	p.(Lys821Glu) (NP_055030.1)	F
17	60003873	C	T	*INTS2*	NM_020748.2:c.157G>A	:p.(Ala53Thr) (NP_065799.1)	F
17	73485444	G	A	*KIAA0195*	NM_014738.4:c.862G>A	p.(Val288Ile) (NP_055553.3)	F
6	138582682	C	T	*KIAA1244*	NM_020340.4:c.1123C>T	p.(Arg375Cys) (NP_065073.3)	F
13	74420487	G	A	*KLF12*	NM_007249.4:c.147C>T	p.(Pro49 = ) (NP_009180.3)	F
6	42986134	C	A	*KLHDC3*	NM_057161.3:c.573C>A	p.(His191Gln) (NP_476502.1)	F
22	29545589	G	A	*KREMEN1*	NC_000022.10(NM_032045.4):c.1416+7501G>A		F
17	79885565	C	G	*MAFG*	NM_002359.3:c.-191G>C		F
19	3786302	A	G	*MATK*	NC_000019.9(NM_002378.3):c.76-1420T>C		F
11	102668089	G	T	*MMP1*	NM_002421.3:c.248C>A	p.(Thr83Asn) (NP_002412.1)	F
1	11307911	A	T	*MTOR*	NM_004958.3:c.1081T>A	p.(Cys361Ser) (NP_004949.1)	F
8	71036930	C	T	*NCOA2*	NM_006540.2:c.4087G>A	p.(Gly1363Arg) (NP_006531.1)	F
4	95496916	G	A	*PDLIM5*	NC_000004.11(NM_001256426.1):c.292-178G>A		F
3	47458897	C	A	*SCAP*	NM_012235.2:c.2867G>T	p.(Gly956Val) (NP_036367.2)	F
1	99127352	G	A	*SNX7*	NM_015976.4:c.65G>A	p.(Gly22Glu) (NP_057060.2)	F
7	48033927	C	T	*SUN3*	NM_152782.3:c.846G>A	p.(Lys282 = ) (NP_689995.3)	F
19	14674625	G	A	*TECR*	NM_138501.*5*:c.177G>A	p.(Leu59 = ) (NP_612510.1)	F
1	92161298	T	A	*TGFBR3*	NM_003243.4:c.2368A>T	p.(Ile790Phe) (NP_003234.2)	F
20	52109752	A	G	*TSHZ2*	NM_173485.5:c.*6078A>G		F
13	115047277	G	T	*UPF3A*	NM_023011.3:163G>T	p.(Gly55Cys) (NP_075387.1)	F
10	75556529	C	T	*ZSWIM8*	NC_000010.10(NM_001242488.1):c.3019-3C>T		F
1	104297389	C	T	*AMY1C*	NM_001008219.1:c.1054C>T(NM_001008219.1)	p.(Arg352Ter) (NP_001008220.1)	G
19	19765499	C	T	*ATP13A1*	NM_020410.2:c.1666G>A	p.(Glu556Lys) (NP_065143.2)	G
19	1237747	G	A	*C19orf26*	NC_000019.9 NM_152769.2:c.-22+8C>T		G
1	75038073	T	-	*C1orf173*	NM_001002912.4:c.3321delA	p.(Glu1108fs) (NP_001002912.4)	G
2	55746980	A	C	*CCDC104*	NM_080667.5:c.43A>C	p.(Ser15Arg) (NP_542398.3)	G
11	68571565	A	G	*CPT1A*	NM_001876.3:c.458T>C	p.(Met153Thr) (NP_001867.2)	G
17	1340295	C	T	*CRK*	NM_016823.3:c.396G>A	p.(Glu132 = ) (NP_058431.2)	G
19	41307024	G	A	*EGLN2*	NM_053046.3:c.547G>A	p.(Val183Met) (NP_444274.1)	G
13	41515331	G	A	*ELF1*	NM_172373.3:c.982C>T	p.(Arg328Trp) (NP_758961.1)	G
17	78395733	C	T	*ENDOV*	NM_173627.3:c.334C>T	p.(Arg112Trp )(NP_775898.2)	G
9	130703472	G	T	*FAM102A*	NM_001035254.2:c.*1999C>A		G
11	64011310	C	T	*FKBP2*	NC_000011.9(NM_004470.3*)*:c.332-3C>T		G
19	48248821	C	T	*GLTSCR2*	NM_015710.4:c.5C>T	p.(Ala2Val) (NP_056525.2)	G
5	90136800	A	G	*GPR98*	NM_032119.3:c.17017A>G	p.(Lys5673Glu) (NP_115495.3)	G
1	156593354	C	T	*HAPLN2*	NM_021817.2:c.72C>T	p.(Ala24 = ) (NP_068589.1)	G
5	177634178	C	G	*HNRNPAB*	NM_031266.2:c.621C>G	p.(Pro207 = ) (NP_112556.2)	G
5	53751640	G	T	*HSPB3*	NM_006308.2:c.21G>T	p.(Arg7Ser) (NP_006299.1)	G
17	1410318	C	G	*INPP5K*	NM_016532.3:c.732G>C	p.(Pro244 = ) (NP_057616.2)	G
8	12879416	C	T	*KIAA1456*	NM_020844.2:c.1228C>T	p.(Arg410Cys) (NP_065895.2)	G
12	25368410	C	T	*KRAS*	NM_033360.2:c.535G>A	p.(Gly179Ser) (NP_203524.1)	G
11	68171104	G	A	*LRP5*	NM_002335.2:c.1738G>A	p.(Val580Ile) (NP_002326.2)	G
19	6212434	C	T	*MLLT1*	NM_005934.3:c.*619G>A		G
2	55476623	G	T	*MTIF2*	NM_002453.2:c.889C>A	p.(Pro297Thr) (NP_002444.2)	G
5	137211606	G	C	*MYOT*	NM_006790.2:c.445G>C	p.(Glu149Gln) (NP_006781.1)	G
12	132633427	C	T	*NOC4L*	NM_024078.1:c.888C>T	p.(Arg296 = ) (NP_076983.1)	G
13	33338714	C	T	*PDS5B*	NM_015032.3:c.3606C>T	p.(Asp1202 = ) (NP_055847.1)	G
6	122931475	G	A	*PKIB*	NC_000006.11(NM_001270394.1):c.-200-22953G>A		G
1	89150050	G	A	*PKN2*	NM_006256.2:c.-214G>A		G
3	129286638	GAC	-	*PLXND1*	NM_015103.2:c.3874_3876delGTC	p.(Val1292del) (NP_055918.2)	G
5	89808335	A	G	*POLR3G*	NM_006467.2:c.*379A>G		G
1	42925741	TT	-	*PPCS*	NM_024664.2:c.*144_*145delTT		G
1	12837669	G	T	*PRAMEF12*	NM_001080830.1:c.1379G>T	p.(Gly460Val) (NP_001074299.1)	G
1	12837720	G	A	*PRAMEF12*	NM_001080830.1:c.1430G>A	p.(Cys477Tyr) (NP_001074299.1:	G
5	139498729	AGAA	-	*PURA*	NM_005859.4:c.*3994_*3997delAGAA		G
1	109780612	C	G	*SARS*	NM_006513.3:c.*102C>G		G
19	4546268	G	A	*SEMA6B*	NM_032108.3:c.1698C>T	p.(Asp566 = ) (NP_115484.2:	G
9	130869703	C	G	*SLC25A25*	NM_001006641.3:c.1492C>G	p.(Leu498Val) (NP_001006642.1:	G
19	56012091	C	T	*SSC5D*	NM_001144950.1:c.2537C>T	p.(Ala846Val) (NP_001138422.1:	G
19	4816902	C	T	*TICAM1*	NM_182919.3:c.1488G>A	p.(Pro496 = ) (NP_891549.1:	G
5	72419666	C	T	*TMEM171*	NM_173490.6:c.466C>T	p.(Arg156Trp) (NP_775761.4:	G
6	116599859	T	C	*TSPYL1*	NM_003309.3:c.1135A>G	p.(Thr379Ala) (NP_003300.1:	G
12	49375692	C	G	*WNT1*	NM_005430.3:c.*269C>G		G
19	37441182	C	T	*ZNF568*	NM_198539.3:c.1127C>T	p.(Ser376Phe) (NP_940941.2:	G
17	42854580	G	A	*ADAM11*	NM_002390.4:c.1728G>A	p.(Thr576 = ) (NP_002381.2:	H
4	88053456	G	T	*AFF1*	NM_001166693.1:c.3207G>T	p.(Met1069Ile) (NP_001160165.1:	H
11	111739334	T	C	*ALG9*	NM_024740.2:c.397A>G	p.? (NP_079016.2)	H
11	116693892	C	T	*APOA4*	NM_000482.3:c.16G>A	p.(Val6Met) (NP_000473.2)	H
17	40970997	G	A	*BECN1*	NC_000017.10 (NM_003766.3*)*:c.261-102C>T		H
17	48653130	G	A	*CACNA1G*	NM_018896.4:c.1367G>A	p.(Arg456Gln) (NP_061496.2)	H
11	34120073	A	G	*CAPRIN1*	NC_000011.9(NM_005898.4*)*:c.2065+765A>G		H
4	110624537	C	T	*CASP6*	NM_001226.3:c.15G>A	p.(Ser5 = ) (NP_001217.2)	H
11	58393171	A	-	*CNTF*	NM_000614.3:c.*1176delA		H
15	33359950	C	G	*FMN1*	NM_001277313.1:c.2044-2675G>C	p.(Glu46 = ) (NP_001096654.1)	H
11	105769010	T	A	*GRIA4*	NM_000829.3:c.742T>A(NM_000829.3)	p.(Ser248Thr) (NP_000820.3)	H
9	5772931	C	T	*KIAA1432*	NM_020829.3:c.3834C>T	p.(Asp1278 = ) (NP_065880.2)	H
11	60160176	C	A	*MS4A7*	NM_021201.4:c.565C>A	p.(Leu189Ile) (NP_067024.1)	H
1	40367533	C	A	*MYCL*	NM_001033082.2:c.28G>T	p.(Ala10Ser) (NP_001028254.2)	H
1	40367535	G	A	*MYCL*	NM_001033082.2:c.26C>T	p.(Ala9Val) (NP_001028254.2)	H
11	69064721	A	G	*MYEOV*	NM_138768.2:c.*862A>G		H
11	66192648	G	A	*NPAS4*	NM_178864.3:c.2287G>A	p.(Ala763Thr) (NP_849195.2)	H
3	136047691	C	T	*PCCB*	NM_001178014.1:c.1550C>T	p.(Ala517Val) (NP_001171485.1)	H
11	65404802	C	T	*PCNXL3*	NM_032223.2:c.*353C>T		H
11	64697864	C	T	*PPP2R5B*	NC_000011.9(NM_006244.3*)*:c.782+11C>T		H
11	64532210	T	C	*SF1*	NM_001178030.1:c.*716A>G		H
3	133748570	G	A	*SLCO2A1*	NM_005630.2:c.77C>T	p.(Ser26Leu) (NP_005621.2)	H
1	59041116	T	C	*TACSTD2*	NM_002353.2:c.*741A>G		H
4	122682720	C	T	*TMEM155*	NM_152399.2:c.185G>A	p.(Arg62His) (NP_689612.2)	H
4	147824789	G	A	*TTC29*	NM_031956.2:c.493C>T	p.(Arg165Ter) (NP_114162.2)	H
11	118951881	C	T	*VPS11*	NM_021729.4:c.2517C>T	p.(His839Tyr) (NP_068375.3)	H

^α^CTGCTGGAGCTGCTGCTGCTGTAA,

^β^CATCCACCCAGACACCCATT,

* 3’UTR

#### Nonsynonymous variants

We conducted the MM-KBAC analysis on 11,272 rare nonsynonymous variants in 4,877 genes and obtained a total of 316 genes with *p*<0.05. After annotation of variants, we obtained 87 variants that co-segregated within families. One variant in *COPZ2* was removed from analysis based on the MAF>0.01 reported in ExAC although it was not reported in the 1000 Genomes data.

#### LoF variants

The analysis was performed on 1,364 rare LoF variants located in 711 genes and a total of 60 genes were obtained with a *p*<0.05. Following annotation, 13 deleterious variants co-segregated within families (Table 2). For Indel variants, BAM files were manually examined using the Genome browser in SVS v8.6 (Golden Helix) to verify the variant.

#### Variants in 5’-UTR and 3’-UTR regions

The MM-KBAC analysis was conducted on 26,872 rare variants in 8,299 genes and 409 genes were obtained with a *p*<0.05. Following annotation of variants and analysis of co-segregation, 25 variants co-segregated within families (Table 2).

#### Synonymous variants

The analysis was performed on 5,854 synonymous rare variants located in 3,164 genes and a total of 216 genes with a *p* value<0.05 were obtained. Following annotation, a total of 35 variants co-segregated within families (Table 2). A variant in *ASB16* was excluded from the analysis based on the allele frequency reported in ExAC (MAF = 0.0278). We also investigated whether synonymous variants were located in splicing enhancer and silencer regions within genes. The variants c.429G>A (NM_006024.6), c.3606C>T, c.1809G>A and c.177G>A were identified in enhancer regions in the *TAX1BP1*, *PDS5B*, *NTN1* and *TECR* genes respectively and c.72C>T (NM_02817.2), c.846G>A (NM_152782.3), and c.861C>T (NM_024830.3) were located in splicing silencer regions in the *HAPLN2*, *SUN3*, and *LPCAT1* genes respectively (Table 3).

**Table 3:** Synonymous variants in enhancer and splicing regions identified in families co-segregating with ET based on MM-KBAC analysis of rare variants.

Chr	Position	REF	ALT	Gene name	Variant Type	Motif seq	Motif type	Chromosome location of motif
7	27,809,333	G	A	*TAX1BP1*	synonymous	GAACT**G**	ESE	chr7:27,809,328–27,809,333
13	33,338,714	C	T	*PDS5B*	synonymous	AGA**C**GA GA**C**GAC A**C**GACT	ESE ESE ESE	chr13:33338711–33,338,716 chr13:33338712–33,338,717 chr13:33338713–33,338,718
17	9,143,279	G	A	*NTN1*	synonymous	AGAA**G**G	ESE	chr17:9,143,275–9,143,280
19	14,674,625	G	A	*TECR*	synonymous	CCT**G**AA CT**G**AAG T**G**AAGG **G**AAGGA	ESE ESE ESE ESE	chr19:14674622–14,674,627 chr19:14674623–14,674,628 chr19:14674624–14,674,629 chr19:14674625–14,674,630
1	156,593,354	C	T	*HAPLN2*	synonymous	**C**CAAGG	ESS	chr1:156,593,354–156,593,359
5	1,477,557	G	A	*LPCAT1*	synonymous	**G**GGGTT	ESS	chr5:1,477,557–1,477,562
7	48,033,927	C	T	*SUN3*	synonymous	TTC**C**TT **C**TTGGG	ESS ESS	chr7:48,033,924–48,033,929 chr7:48,033,927–48,033,932

#### Intronic variants

The MM-KBAC analysis was conducted on 1,174,082 intronic rare variants located in 16,486 genes and 324 genes with a *p* value<0.05 were obtained. Following annotation and co-segregation analysis, we obtained a total of 14 deleterious variants that co-segregated within families (Table 2).

#### DNAse I hypersensitivity sites and transcription factor binding sites

Genetic variants can affect transcription factor binding sites (TFBS), particularly via their enrichment in *DNase I* hypersensitive sites (DHS) that provide open chromatin access to transcription factors. Thus we sought variants that could be enriched at these sites using TFBS conserved data in ENCODE [46]. We asked whether the 169 variants (MM-KBAC analysis by variant type, and that includes annotated variants that co-segregated within ET families) identified from our analyses were found in DHS. 67 variants in 65 genes were in DHS. These 67 variants comprised 6 of 67 (9%) 5’-UTR variants; 6 of 67 (9%) 3’-UTR variants; 3 of 67 (4%) were LoF variants; 36 of 67 (54%) were nonsynonymous variants; 12 of 67 (18%) were synonymous; and 4 of 67 (6%) intronic variants.

DHSs are enriched with transcription factor binding sites (TFBSs), crucial sequences for the regulation of gene expression. Cross species conservation of genomic sequence has been successfully used for identifying biologically functional TFBS [47]. We identified 40 variants within TFBS (Table 4).

**Table 4:** Variants located within TFBS identified in families co-segregating with ET.

Chr	Position	Reference	Alternates	Transfac binding matrix id	Strand	Family
1	11307911	A	T	TCF11MAFG_01	+	F
1	40367533	C	A	ELK1_01	+	H
1	40367535	G	A	ELK1_01	+	H
1	92161298	T	A	CART1_01	-	F
2	70524477	G	C	CREB_02	+	C
3	47458897	C	A	MAZR_01	+	F
3	135721907	A	G	YY1_01	-	C
3	136047691	C	T	LUN1_01	+	H
4	88053456	G	T	YY1_01	-	H
4	95496916	G	A	PAX4_04	+	F
5	53751640	G	T	HTF_01	+	G
5	72419666	C	T	SEF1_C	-	G
5	90136800	A	G	MEF2_04	+	G
6	42146612	A	G	COMP1_01	+	F
6	42986134	C	A	HOX13_01	+	F
6	122931475	G	A	SP1_Q6	+	G
8	23177415	C	G	AHRARNT_02	+	B
8	53321917	C	T	AREB6_01	-	A
8	71036930	C	T	AREB6_04	+	F
11	60160176	C	A	NRSF_01	-	H
11	62283386	A	C	HNF1_01	+	A
11	66192648	G	A	AREB6_04	-	H
11	68171104	G	A	TCF11_01	+	G
11	69064721	A	G	TBP_01	+	H
11	75167849	AT	-	PPARA_01	-	F
11	102668089	G	T	AREB6_04	+	F
13	74420487	G	A	SRF_01	-	F
14	103404716	C	T	P53_01	+	F
17	1410318	C	G	PAX3_01	-	G
17	7722271	G	A	CREB_02	+	F
17	42854580	G	A	PAX5_01	+	H
17	43922409	A	G	TAXCREB_01	-	C
17	73485444	G	A	NRSF_01	-	F
17	79885565	C	G	AP4_01	-	F
17	80918994	C	T	PAX4_01	-	F
18	55362414	-	A	TCF11_01	-	C
19	1237747	G	A	PAX5_01	-	G
19	4816902	C	T	HEN1_01	+	G
19	19765499	C	T	PPARA_01	-	G
19	48248821	C	T	YY1_02	-	G

### Phenolyzer analysis

We used phenolyzer to prioritize candidate genes within ET families. The results of the phenolyzer network analysis for 5 families (A, B, D, F, H) are shown in S2 Fig.

#### Family A

*KARS* is predicted to be the most disease relevant seed gene (raw score 0.03532; normalized score 0.004) because it maps to Charcot Marie Tooth disease recessive intermediate b in OMIM (OMIM 613641) and DISGENET (C3150897)(S2 Fig). The nonsynonymous variant identified in *KARS* (c.1513C>T (NM_001130089.1), p.(Arg505Cys)) has a Phred scaled CADD score of 28.6 and is predicted to be deleterious or damaging by several *In Silico* tools (SIFT, POLYPHEN2, Mutation Taster, FATHMM, Provean, MetaSVM and Meta LR). The top three predicted genes are *ARGEF1* (normalized score 0.011), *PHOSPHO1* (normalized score 0.008) and *AMBRA1* (normalized score 0.004).

#### Family B

*KIF5A* is predicted to be the most disease relevant seed gene (raw score 0.2954; normalized score 0.033) because it maps to spastic paraplegia 10 in OMIM (OMIM 604187) and DISGENET (C1858712). The variant identified in *KIF5A* is a synonymous variant (c.2769G>A (NM_004984.2), p.(Arg923 = )) with a phred-scaled CADD score of 10.95. The nucleotide c.2769 (NM_004984.2) (Chr12:57,975,211) is highly evolutionarily conserved and the FAS-ESS web tool identifies the exon splicing motif ‘CCACTA’ in close proximity (Chr12:57,975,217–57,975,222). The top four predicted genes include *ARHGEF28* (raw score 0.1506; normalized score 0.016), *PSD4* (raw score 0.1208; normalized score 0.013), *LPCAT1* (raw score 0.09227; normalized score 0.01) and *KCNH3* (raw score 0.08023; normalized score 0.008) based on their protein interactions, the same biosystem (e.g. *ARHGEF28*, biosystem Axon guidance, EH-Ephrin signaling and developmental biology), the same gene family (e.g. *PSD4*; gene family, Pleckstrin homology (PH) domain containing or *KCNH3*; gene family, potassium channels Voltage-gated ion channels) or transcription interactions (e.g. *LPCAT1* regulated by ETS1 transcription factor).

#### Family C

The top ranked gene is a predicted gene, *MATK* (raw score 0.4266; normalized score 0.046) based on protein interactions (e.g. yeast 2-hybrid with *EWSR1*), the same biosystem (e.g. signal transduction, neurotrophic factor-mediated Trk receptor signalling), the same gene family (e.g. SH2 domain containing) or transcription interactions (e.g. regulated by *GATA2*). The next top three genes (predicted) are *WNT9B* (normalized score 0.025), *TAX1BP1* (normalized score 0.015) and *PPP2R3A* (normalized score 0.015).

#### Family D

*SLIT3* is predicted to be one of the most disease relevant seed gene, with a raw score of 0.1637 and normalized score of 0.017, respectively (S2 Fig). *SLIT3* maps to temporal lobe epilepsy in DISGENET (C0014556) but a disease association with *SLIT3* has not been described in OMIM. The non-synonymous variant identified in *SLIT3* (c.3505G>C (NM_003062.3), p.(Val1169Leu)); rs144799628) has a Phred scaled CADD score of 22.5 and is predicted to be deleterious or damaging by several *in silico* tools (LRT Pred, Mutation Taster, and FATHMM). The top three predicted genes are *TRAF2* (normalized score 0.035), *EPHB3* (normalized score 0.016) and *SLC22A16* (normalized score 0.01). The variants identified in *TRAF2* (c.1335C>T (NM_021138.3), p.(Asp445 = )); phred scaled CADD score of 10.96) and *EPHB3* (c.618C>T (NM_004443.3), p.(Arg206 = )); phred scaled score 13.71) are synonymous variants with weak evidence for pathogenicity. The *SLC22A16* (also known as *OCT6*) variant (c.1309delC (NM_033125.3), p.(Gln437fs)) is a LoF frameshift variant, with a phred-scaled CADD score of 35, that is predicted to result in premature termination of the protein.

#### Family E

No annotated (phred-scaled CADD score >10 or predicted deleterious or damaging by *in silico* tools) segregating rare deleterious variants were identified in Family E.

#### Family F

The top predicted disease relevant seed gene is *NTRK1* (raw score 5.152; normalized score 0.538) based on disease mapping to congenital sensory neuropathy with anhidrosis, hereditary sensory and autonomic neuropathy IV (HSAN4) and familial dysautonomia type II in OMIM (OMIM 256800), DISGENET (C0020074), and ORPHANET (642). The variant identified in *NTRK1* is an intronic variant (intron 2;NM_001007792.1:c.122+2627T>C) located in an ENCODE annotated open chromatin/TFBS region (openChrom_2127) of the *NTRK1* gene. The top three predicted genes are *GIPC1* (normalized score 0.06), *MATK* (0.045) and *NCOA2* (normalized score 0.04).

#### Family G

The top ranked and predicted seed gene is *CRK* (raw score 0.6991; normalized score 0.073) based on disease mapping in DISGENET, protein interactions (PUBMED 16713569; yeast 2-hybrid with *ATXN1*, score 0.004856), the same biosystem (e.g. signal transduction; *NGF* signaling via *TRKA* form the plasma membrane; signal transduction; signalling to ERKs; signalling by *NGF*; neurotrophic factor-mediated Trk receptor signaling), the same gene family (e.g. SH2 domain containing) or transcription interactions.

#### Family H

*CACNA1G* is predicted to be the most disease relevant seed gene (raw score 0.3719; normalized score 0.039) because it maps to Spinocerebellar ataxia 42 in OMIM (OMIM 616795) (S2 Fig). The nonsynonymous variant identified in *CACNA1G* (c.1367G>A (NM_018896.4), p.(Arg456Gln)) has a phred-scaled CADD score of 16.13 and is predicted to be deleterious or damaging by several *In Silico* tools (POLYPHEN2, Mutation Taster, FATHMM, Provean, MetaSVM and Meta LR). The top three predicted genes are *PPP2R5B* (intronic variant; normalized score 0.4464), *CASP6* (synonymous variant; normalized score 0.021) and *ADAM11* (synonymous variant; normalized score 0.016).

#### CACNA1G

We evaluated all candidate genes prioritized by phenolyzer in a previously published WES dataset of ET families [15]. We identified two additional families (S3 Fig). One family had a non-synonymous variant in *CACNA1G* (c.3635G>A (NM_018896.4), p.(Arg1212Gln)), rs150972562) that is highly conserved evolutionarily and is predicted to be deleterious or damaging by several *in silico* tools (provean (score: -3.62), SIFT (score: 0.002) and Mutation Taster (disease causing)) that co-segregated with ET. The second family, also had a non-synonymous variant in *CACNA1G* (c.1879G>A (NM_018896.4), p.(Gly627Arg), that is highly conserved evolutionarily (GERP score: 5.7199) and is predicted to be damaging (DANN score: 0.9977) that co-segregated with ET. These *CACNA1G* variants were apparent retrospectively but was not identified in the prior analysis using the bioinformatics pipeline or analysis methods applied in the WES study. The allele frequency of rs150972562 in the Genome Aggregation Database (gnomAD) database is 0.001473 (413/280340+1 homozygote), and for the c.1879G>A variant is 0.001356 (308/227140+1 homozygote) which is below the estimates of the disease prevalence of ET at 2–4%.

### Rare CNVs segregating with ET in families

We detected a total of 7 rare genic CNVs that segregated with ET in 5 families (Families A, C, F, G and H)(Table 5). Four CNVs were copy number gains and three were deletions. The size of CNVs ranged from ~4Kb to 17Kb. We did not identify rare genic CNVs >100Kb that segregated with ET in families. CNVs in known ET associated genes (e.g. *LINGO1*) or other neurodegenerative genes were not identified. In family A, a 4.8Kb deletion in intron 2 of the *GUCY1A3* gene was identified in addition to two duplications in intron 2 of *KANSL1*. The duplications in *KANSL1* span ENCODE annotated H3K27AC marks, DNAse hypersensitive clusters and transcription factor binding sites that are often found near regulatory elements and promoters. In family C, a 3.9kb deletion spanning DNase hypersensitive clusters ~10kb downstream of *SOD2* was identified. In family F a 17.3kb deletion spanned several genes including *CDK11A*, *CDK11B*, *AK097814*, *intron 5/exon 6 of SLC35E2A and intron 1 of SLC32E2B*. Intronic duplications in *TAOK1* and *C1ORF185* were identified in families G and H respectively.

**Table 5:** Rare CNVs segregating with ET in families.

Family ID	Type	Copy number (CN)	Cytoband	Genomic coordinates GRCh37/hg19	Size (kb)	Gene or closest gene	Intron/exon	Omim #	Gene function
A	Loss	CN = 1	4q32.1	chr4:156,593,436–156,598,228	4.8	*GUCY1A3*	Intron 2	139396	Catalyzes conversion of GTP to cGMP. Functions as main receptor for NO
A	Gain	CN = 3	17q21.31	chr17:44,212,968–44,226,250	13.3	*KANSL1*	Intron 2	610443	Role in chromatin modification. Member of histone acetyltransferase complex
A	Gain	CN = 3	17q21.31	chr17:44,285,463–44,294,088	8.6	*KANSL1*	Intron 2	610443	Role in chromatin modification. Member of histone acetyltransferase complex
C	Loss	CN = 1	6q25.3	chr6:160,085,604–160,089,457	3.9	*SOD2* nearest gene	downstream region	147460	Mitochondrial matrix enzyme. Scavenges ROS
F	Loss	CN = 1	1p36.33	chr1:1,648,775–1,666,096	17.3	*CDK11A/B* *SLC35E2A* *SLC35E2B* *AK097814*	*CDK11A/B*: intron/exons 1–5 *SLC35E2A*: exon 6 intron 5 *SLC32EB*: intron 1 A *AK097814*:intron/exons 1–2	NA	CDK11A/B-Serine threonine protein kinase that can be cleaved by caspases and may play a role in cell apoptosis SLC35E2A/B:antiporter activity. Transmembrane transporter activity AK097814-unknown
G	Gain	CN = 3	17q11.2	chr17:27,811,701–27,815,623	3.9	*TAOK1*	Intron 8	NA	Serine/threonine protein kinase involved in p38/MAPK14 stress-activated MAPK cascade, DNA damage response and regulation of cytoskeleton
H	Gain	CN = 3	1p32.3	chr1:51,589,476–51,600,924	11.4	*C1ORF185*	Intron 3	NA	Unknown

### Functional studies

#### *Slit Drosophila* model and nervous system dysfunction

**Fig 3:**
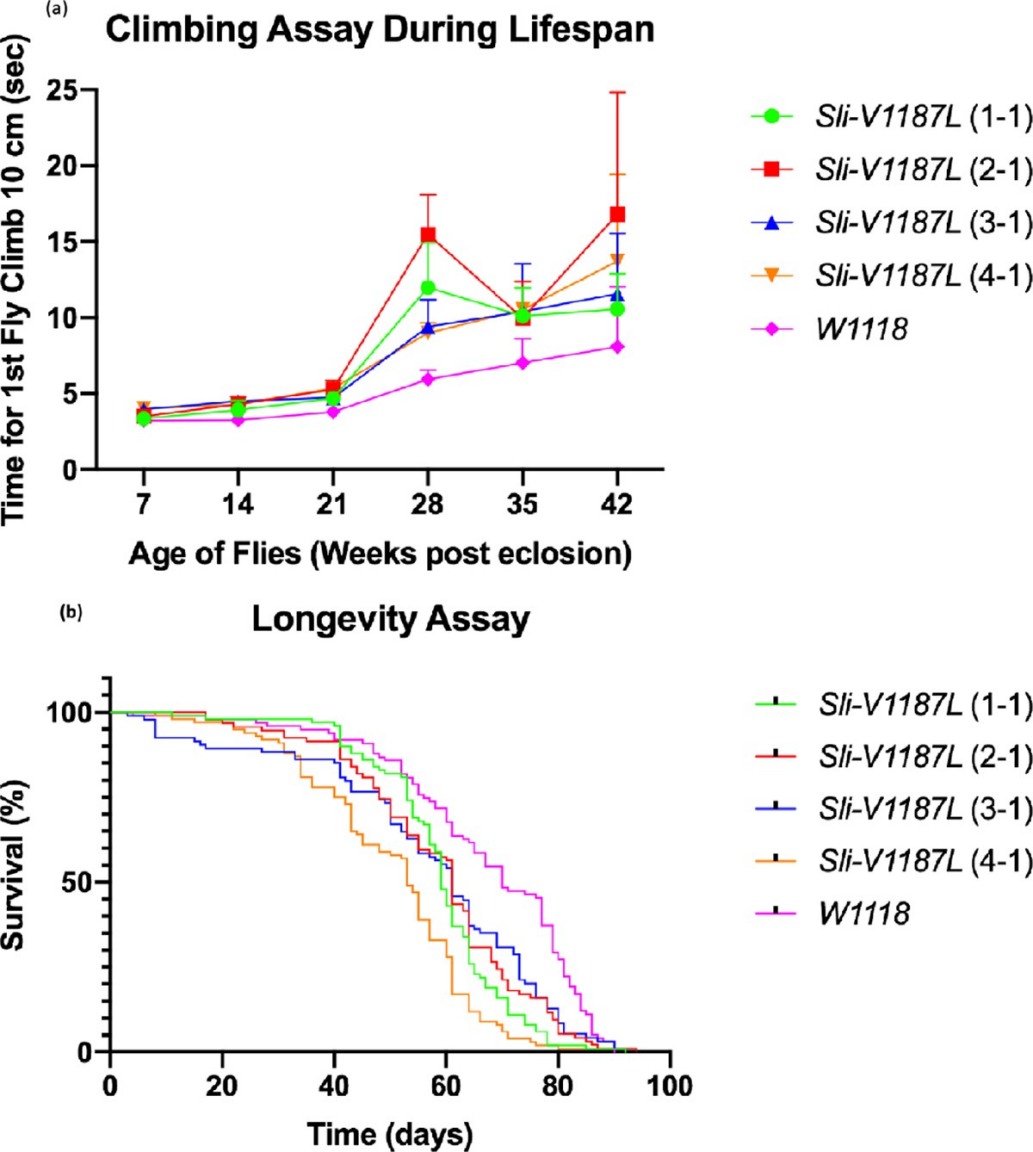
Behavioral manifestations of nervous system dysfunction in a *slit Drosophila* model. (a) Climbing response during lifespan. The climbing assay was assessed as the time taken for the first fly to climb 10.0cm. The mean climbing index + SEM as a function of age is shown for each independent mutant *slit* line and the wildtype line. Each point represents the mean of 10 flies. Flies expressing the mutant slit (p.Val1187Leu) compared to wildtype slit displayed significantly slower climbing (*p*<0.05) throughout lifespan. (b) Survival assays in *slit* lines. A total of 100 virgin flies per line were sex segregated within 4h of eclosion and maintained in small laboratory vials (n = 20 per vial) containing fresh food in a low-temperature incubator at 25°C and 40% humidity on a 12/12h dark/light cycle. The flies were transferred to fresh food vials every 3–4 days and mortality recorded. Significant differences in lifespan were detected between flies expressing the mutant slit (p.Val1187Leu) compared to wildtype slit (*p*<0.0001).

To determine the pathogenicity of the *SLIT3* variant (c.3505G>C (NM_001271946.1), p.(Val1169Leu)(*Drosophila slit* p.Val1187Leu; NP_001261017.1) that we identified in family D, *Drosophila slit* lines were created. To test the hypothesis that the *slit* p.Val1189Leu variant causes nervous system dysfunction we evaluated climbing response throughout the fly lifespan. Flies expressing the mutant slit (p.Val1187Leu) compared to wildtype slit displayed significantly slower climbing (*p*<0.05) throughout lifespan (Fig 3) suggesting that the *slit* p.Val1187Leu mutation causes age related locomotor changes. Because ET is associated with increased mortality we performed survival assays. Significant differences in lifespan were detected between flies expressing the mutant slit (p.Val1187Leu) compared to wildtype slit (*p*<0.0001) suggesting that the slit p.Val1187Leu mutation is associated with reduced lifespan (Fig 3).

#### Ca_V_3.1 electrophysiology

**Fig 4:**
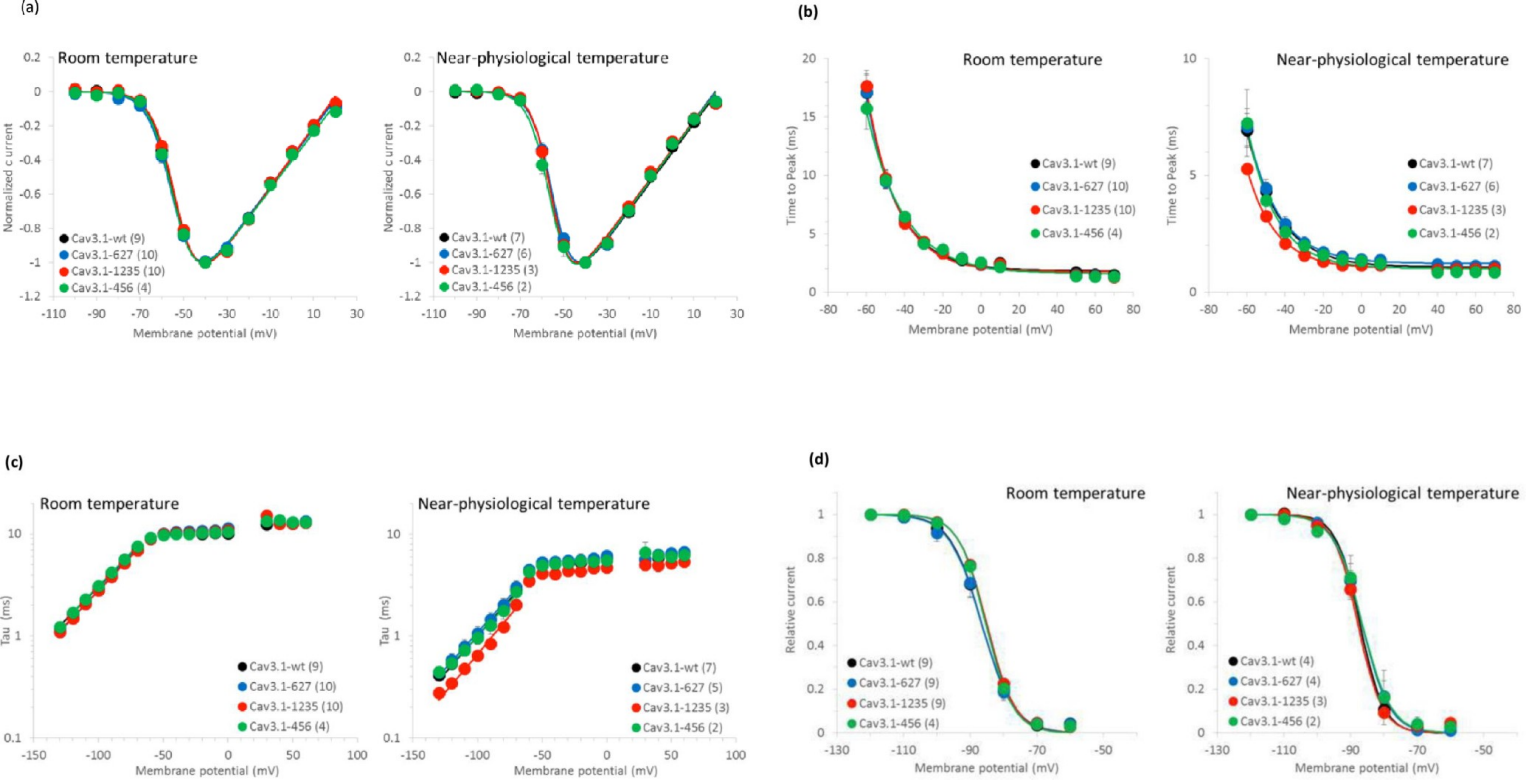
Ca_v_3.1 electrophysiology for mutant and wildtype channels at room temperature and at near physiological temperature. (a) Current-Voltage relationship of wildtype and mutant Ca_v_3.1 channels. (b) Time to peak with wild type and mutant Ca_v_3.1 channels. (c) I-V relationship of wild type and mutant Ca_v_3.1 channels. (d) Steady state inactivation of wild type and mutant channels.

To determine the functional effects of *CACNA1G* variants identified in ET families, electrophysiology studies by whole cell patch clamp recordings was performed in HEK293T cells expressing the Ca_V_3.1 mutant channels. At room temperature or at near physiological temperatures the current voltage relationship and the time measured from the beginning of the depolarizing pulse to the peak of the inward current (time to peak) (Fig 4) observed in mutant and wild type channel variants showed no significant differences. Kinetics of channel closing (time constant of deactivation and inactivation) and steady state inactivation (Fig 4) were similar between wild type and mutant channel variants. The voltage dependence of Ca_V_3.1–1235 channel activation showed a trend in altered channel function with a small shift to more negative values (Fig 4) and channel deactivation was shifted to positive values (Fig 4).

## Discussion

In this study, we applied the MM-KBAC test [19] to analyze rare SNVs and two SV and CNV-detection algorithms Canvas version 1.19.1 and Genome STRucture in Populations (Genome STRiP) version 2.0 in the WGS data generated from eight early-onset ET families enrolled in the family study of Essential Tremor (FASET). While numerous methods have been described for rare variant analysis in case-control studies, relatively few methods exist for family-based studies. The advantages of family-based studies are their robustness to population stratification [48], and the use of information about transmission of genetic factors within families, which is more powerful than population-based case control studies [49]. Genes identified by MM-KBAC analysis in ET families were prioritized using phenolyzer. Phenolyzer prioritizes candidate genes based on disease or phenotype information. Phenolyzer includes multiple components, including a tool to map the user-supplied phenotype to related disease, a resource that integrates existing knowledge on disease genes, an algorithm to predict previously unknown disease genes, a machine learning model that integrates multiple features to score and prioritize candidate genes and a network visualization tool to examine gene-gene and gene-disease relationships [39]. Previously, we performed WES [15] on a subset of the families (Families A, B and F) included in the current WGS analysis. For these families, WES did not identify the candidate genes identified in the current WGS study. There are several reasons why variants and candidate genes could have been missed in the prior WES analysis. Recently published studies [18, 50] suggest that WGS is more powerful than WES for detecting potential disease-causing mutations within WES regions, particularly those due to SNVs. WGS which forgoes capturing is less sensitive to GC content and is more likely than WES to provide complete coverage of the entire coding region. Other factors that can affect variant and candidate gene identification include the bioinformatics pipeline (GATK version and implementation options) used and statistical analysis methods (WES study pVAAST [15, 51] versus WGS in the current study: MM-KBAC [51]). Although, WGS is currently more expensive than WES (WES 50x coverage, Illumina NovaSeq 6000 = $600/sample versus WGS 30x coverage, Illumina HiSeq X = $1,575/sample; price includes library preparation, sequencing, QC of data and bioinformatics and short term data storage) advantages include the capture of non-coding (intronic and intergenic) and regulatory (5’UTR, 3’UTR, promoter and enhancers) variants in addition to coding variants and analysis of structural variants (SVs) and copy number variants (CNVs). Rare genic CNVs that segregated with ET were identified in 5/8 (62.5%) families. Recently, we reported copy number variation of *LINGO1*, a susceptibility gene for ET, in a large 5 generation South Indian family with upper limb postural tremor (dystonic tremor)[52]. We did not identify CNVs in *LINGO1* or other known neurodegenerative genes in the ET families in the current study. Additional studies will be needed to determine the significance of the CNVs identified in the ET families in the current study.

In the current study, within each ET family, we generated a prioritized candidate gene list that can be considered for functional studies. In family H, *CACNA1G* is predicted to be the most disease relevant seed gene because it maps to Spinocerebellar ataxia 42 (SCA42) in OMIM (OMIM 616795). *CACNA1G* is also a genetic modifier of epilepsy [53, 54]. The identification of two additional families, with a deleterious/damaging *CACNA1G* variants, from a previously published WES dataset strongly suggests that *CACNA1G* may be a susceptibility gene for ET. SCA42 is an autosomal dominant neurologic channelopathy disorder characterized predominantly by gait instability, tremor (i.e. intention, postural, head, and resting) and additional cerebellar signs (i.e. dysarthria, nystagmus and saccadic pursuits), and is caused by a heterozygous mutation in *CACNA1G*. There is variable age at onset (range 9- >78 years) and slow progression of the disease. We reviewed the clinical data in the *CACNA1G* families for the characteristic signs of SCA42 including ataxia, gait instability and ocular signs [55–57]. None of the individuals with ET in these families exhibited these problems, suggesting that these families do not have SCA. On the other hand, neuropathologic studies available for an 83 year old affected individual with SCA42, who also had dementia, showed cerebellar atrophy with Purkinje cell loss and loss of neurons in the inferior olive [55], which in terms of the Purkinje cell loss, is consistent with neuropathological findings of some ET patients [58].

The *CACNA1G* gene encodes the pore forming subunit of T-type Ca(2+) channels, Ca_V_3.1, and is expressed in various motor pathways and may serve different functions [59]. The T-type calcium channel, Ca_v_3.1, has been previously implicated in neuronal autorhythmicity [60, 61] and is thought to underlie tremors seen in Parkinson’s disease [62], enhanced physiological tremor, and in ET [63] and T-type calcium channel antagonists have been shown to reduce tremor in mouse models of ET [61, 64, 65].

The identification of *CACNA1G* in three ET families in the current study is consistent with recent reports of mutations in other ion channel genes in other ET families and the concept that the ETs are channelopathies [14, 15]. Electrophysiology studies of the Ca_V_3.1 mutant channels identified in ET families in the current study either showed small differences or no change compared to the wild type channel. The lack of significant differences may reflect the small sample size (number of cells sampled) and that the study was underpowered. However, the voltage dependence of Ca_V_3.1–1235 channel activation showed a trend in altered channel function with a small shift to more negative values and channel deactivation was shifted to positive values. The other two Ca_V_3.1 mutant channels identified in this study are located in the I-II loop of the Ca_V_3.1 channel which is associated with channel trafficking [66] and these variants may effect trafficking to the membrane or cytosolic organelles.

We previously reported the identification of a mutation in *Kv9*.*2* (*KCNS2*), that encodes an electrically silent voltage-gated K^+^ channel α subunit, in a family with pure ET [15]. Kv9.2 is highly and selectively expressed in the brain and modulates the activity of Kv2.1 and Kv2.2 channels, which play a major role in membrane excitability and synaptic transmission and is critical for motor control and other neuronal network functions [67]. In two families with atypical ET, mutations were also identified in genes encoding voltage-gated sodium channel alpha subunits. In a family with epilepsy and ET, a disease-segregating mutation p.(Gly1537Ser) in the *SCN4A* gene was identified and functional analyses demonstrated more rapid channel kinetics and altered ion selectivity, which may contribute to the phenotype of tremor and epilepsy in this family [14]. In a four generation Chinese family, with early onset familial episodic pain and ET, a gain-of-function missense mutation p.(Arg225Cys) in *SCN11A* was identified [68]. Collectively, identification of mutations in a T type Ca(2+) channel (*CACNA1G*; three families, this study), a voltage-gated K^+^ channel α subunit (*Kv9*.*2*; *KCNS2*, 1 family), and voltage-gated sodium channel alpha subunits (*SCN4A* and *SCN11A*) in ET families (five total to date) is emerging evidence that problems in regulation of membrane excitability and synaptic transmission, which are important more broadly for motor control and other neuronal network functions, could play a role in the pathophysiology of ET. The genetic basis of ET has so far remained elusive. Given the clinical and genetic heterogeneity observed in ET [11–16], evaluation of ion channel genes as candidate genes for ET is warranted.

In family D, *SLIT3* is predicted to be the most disease relevant gene. A disease association with *SLIT3* in OMIM has not been described. The non-synonymous variant identified in *SLIT3* (c.3505G>C, p.(Val1169Leu); rs144799628) is highly conserved evolutionarily, is predicted to be deleterious or damaging by several *in silico* tools and has an allele frequency in the ExAC database of 0.0006407 (72/112370+2 homozygotes), which is below the estimates of the disease prevalence of ET at 2–4%. A disease association of SNPs in the *SLIT3* gene and genetic risk (models: susceptibility, survival and age-at-onset) for Parkinson disease was previously identified in two independent GWAS datasets [69]. Axon guidance pathway molecules are involved in defining precise neuronal network formation during development and in the adult central nervous system play a role in the maintenance and plasticity of neural circuits. The Slit axon guidance molecules and their receptors, known as Robo (Roundabout) serve as a repellent to allow precise axon pathfinding and neuronal migration during development. Three Slit ligands have been identified in vertebrates with spatio-temporal expression patterns in the nervous system as well as in the peripheral tissue and other organs during development. Slit or Robo null gene animal models (*Drosophila* or mouse) show that Slit-Robo interactions act as a repulsive signal to regulate actin dynamics for axon guidance at the midline for commissural, retinal, olfactory, cortical and precerebellar axons [70]. The mechanism by which *SLIT3* contributes to ET may involve early degenerative changes in the years preceding diagnosis and possibly even during brain development (the miswiring hypothesis). In one published study, the candidate gene, *TENM4*, which is a regulator of axon guidance and central myelination, was identified in three ET families [12]. This finding together with the identification of *SLIT3* as a candidate gene in an ET family in the current study suggests that in some instances ET may be a disorder of axon guidance. To determine the pathogenicity of the *SLIT3* variant that we identified in family D, *Drosophila slit* lines were created. Behavioral manifestations of nervous system dysfunction were observed in the *Drosophila slit* model suggesting a role in ET disease pathogenesis. Further characterization of the *Drosophila* model will be needed to determine the disease mechanism.

In three families, phenolyzer prioritized genes that are associated with hereditary neuropathies (family A, *KARS*, Charcot-Marie-Tooth disease B (OMIM 613641); family B, *KIF5A*, spastic paraplegia 10 with or without peripheral neuropathy (OMIM 604187); and family F, *NTRK1*, hereditary sensory and autonomic neuropathy IV (OMIM 256800). Among the clinical features of CMTRIB with peripheral neuropathy, electrophysiologic studies show motor nerve conduction velocities of 39.5 and 30.6 m/s in the median and ulnar nerves, respectively consistent with an intermediate phenotype between that of demyelinating and axonal CMT [71]. Heterozygous pathogenic mutations in *KIF5A* are also known to cause an axonal CMT subtype [46]. Interestingly, tremor is known to occur in patients with neuropathies although its reported prevalence varies widely [72]. In a case control study that assessed the presence and severity of tremor using the Fahn-Tolosa-Marin Scale, Archimedes spirals and Bain and Findley spiral score, in 43 consecutively recruited patients with inflammatory neuropathies, twenty seven (63%) patients had tremor (posture or action) with a mean age at tremor onset of 57.6 (11.6) years (widely) [72].

In summary, WGS analysis identified candidate genes for ET in 5/8 (62.5%) of the families analyzed. WES analysis of these families in our previously published study failed to identify candidate genes. Functional studies of two candidate genes identified, *CACNA1G* and *SLIT3*, suggest a role for these genes in ET disease pathogenesis.

The genes and pathways that we have identified can now be prioritized to further our understanding of the pathophysiology of ET using cellular and animal models.

## Supporting information

S1 Fig Sequencing chromatograms showing the point mutation *Drosophila* slit lines (p.Val1187Leu; NP_001261017.1). (PPTX)

S2 Fig Phenolyzer network analysis of WGS gene findings, disease terms and disease types. (PPTX)

S3 Fig Pedigrees for families with *CACNA1G* variant (c.3635G>A (NM_018896.4), p.(Arg1212Gln)) and *CACNA1G* variant (c.1879G>A (NM_018896.4), p.(Gly627Arg)). (DOCX)

S1 Table **Variants identified in families A, B and F in WGS and WES datasets**.
(DOCX)

S2 Table **Variants called in the WGS dataset but not the WES dataset in families A, B and F**.
(DOCX)
