# Supplementary figures and images for: Whole genome sequencing and rare variant analysis in essential tremor families
Source: PLoS One. 2019 Aug 12;14(8):e0220512. doi: 10.1371/journal.pone.0220512 (PMC6690583; doi:10.1371/journal.pone.0220512)

## Slide 1
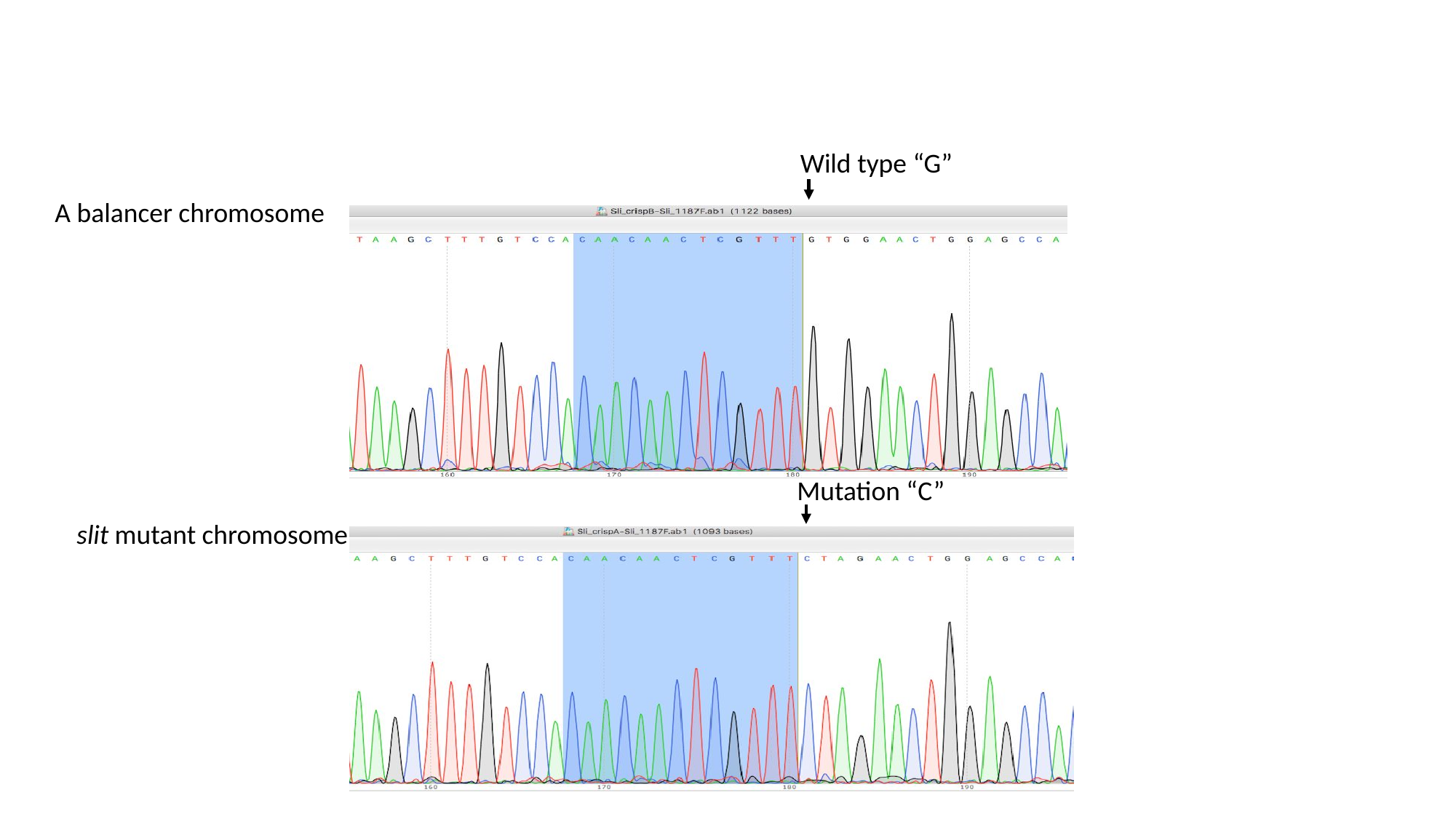

Wild type “G”
A balancer chromosome
Mutation “C”
slit mutant chromosome

Supplement: S1 Fig — (PPTX) [file pone.0220512.s001.pptx]
