# Supplementary material for: Whole genome sequencing and rare variant analysis in essential tremor families
Source: PLoS One. 2019 Aug 12;14(8):e0220512. doi: 10.1371/journal.pone.0220512 (PMC6690583; doi:10.1371/journal.pone.0220512)
Supplement: S2 Fig — (PPTX) [file pone.0220512.s002.pptx]

## Slide 1
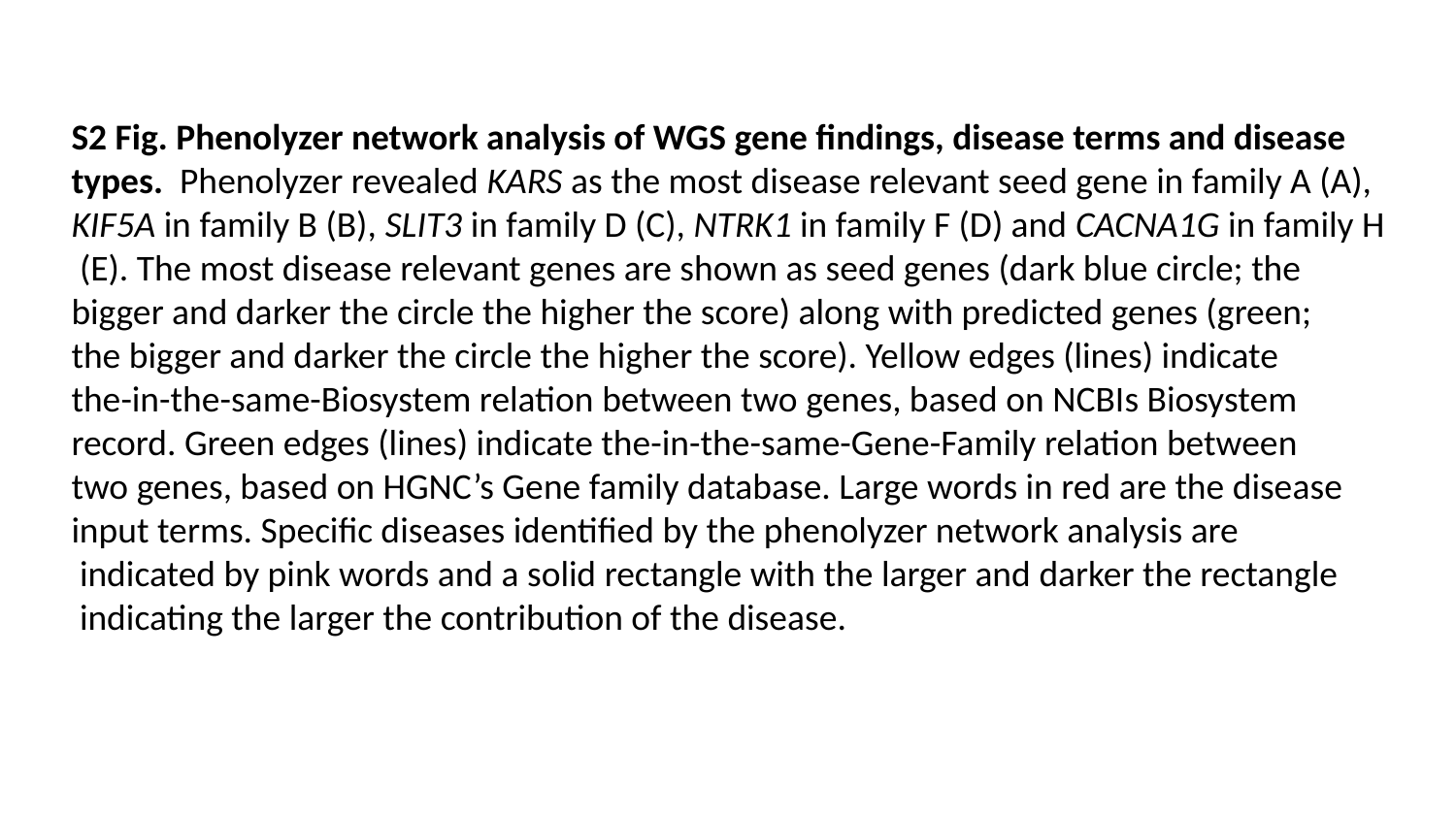

S2 Fig. Phenolyzer network analysis of WGS gene findings, disease terms and disease
types. Phenolyzer revealed KARS as the most disease relevant seed gene in family A (A),
KIF5A in family B (B), SLIT3 in family D (C), NTRK1 in family F (D) and CACNA1G in family H
 (E). The most disease relevant genes are shown as seed genes (dark blue circle; the
bigger and darker the circle the higher the score) along with predicted genes (green;
the bigger and darker the circle the higher the score). Yellow edges (lines) indicate
the-in-the-same-Biosystem relation between two genes, based on NCBIs Biosystem
record. Green edges (lines) indicate the-in-the-same-Gene-Family relation between
two genes, based on HGNC’s Gene family database. Large words in red are the disease
input terms. Specific diseases identified by the phenolyzer network analysis are
 indicated by pink words and a solid rectangle with the larger and darker the rectangle
 indicating the larger the contribution of the disease.

## Slide 2
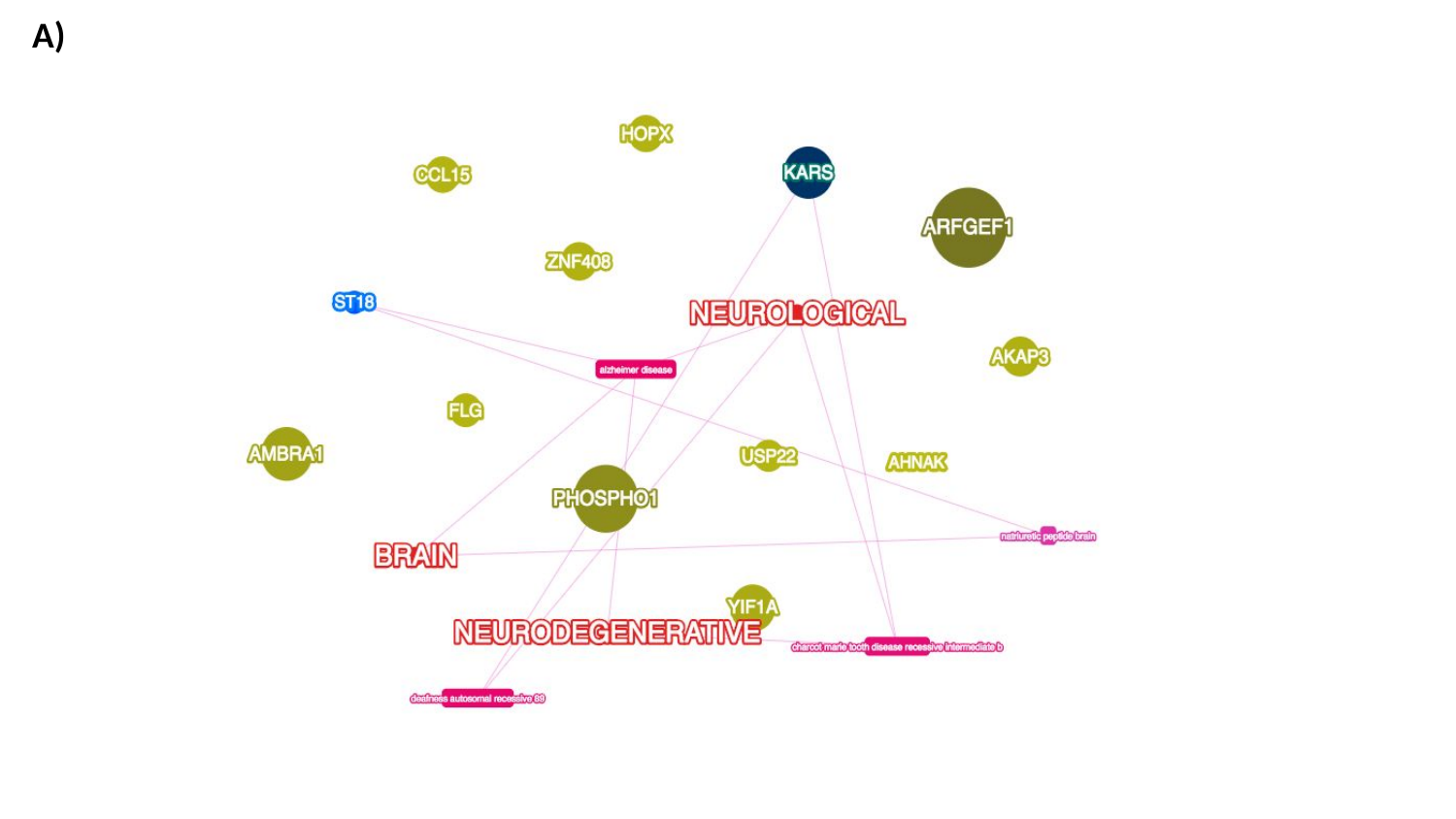

A)

## Slide 3
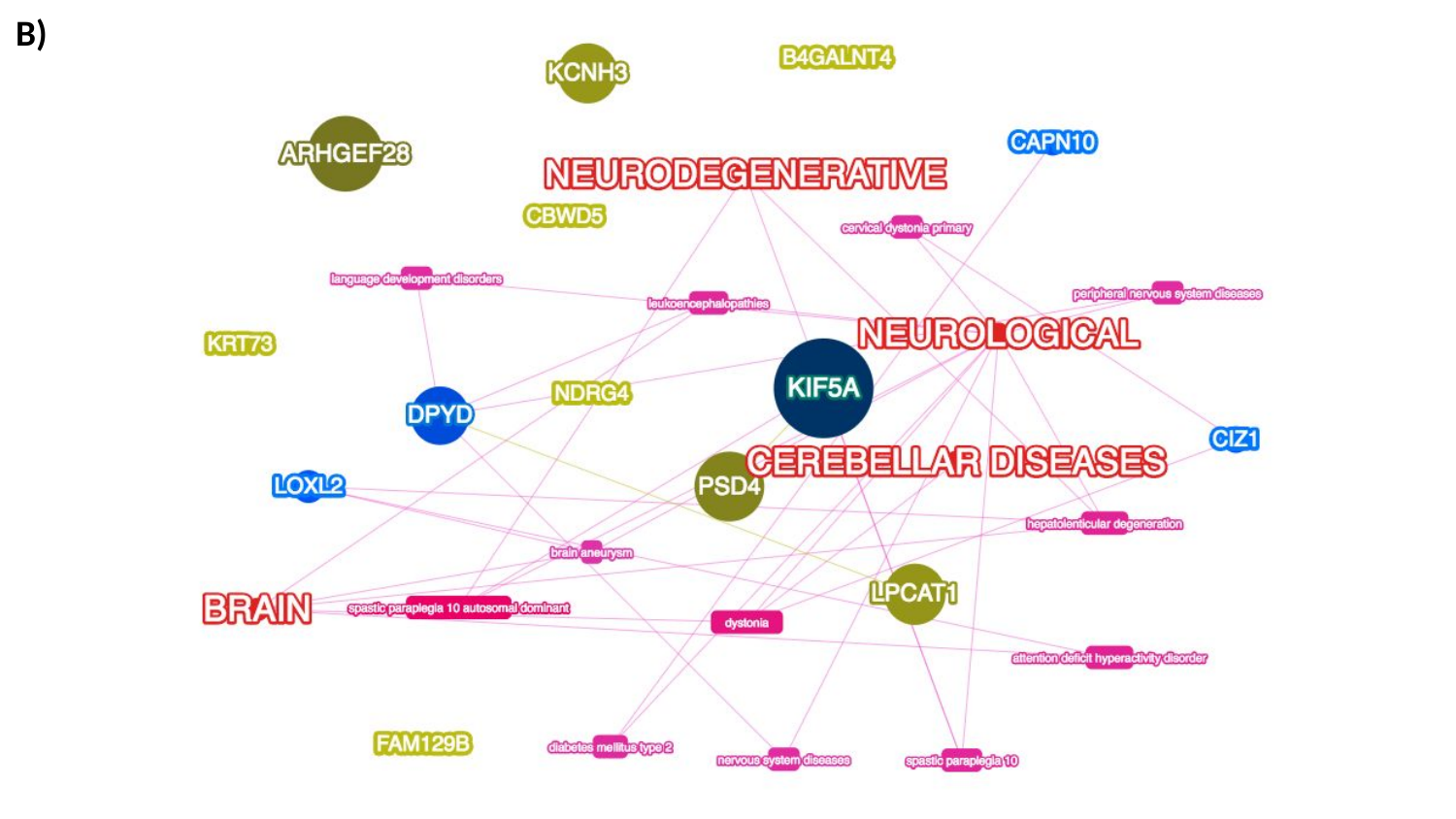

B)

## Slide 4
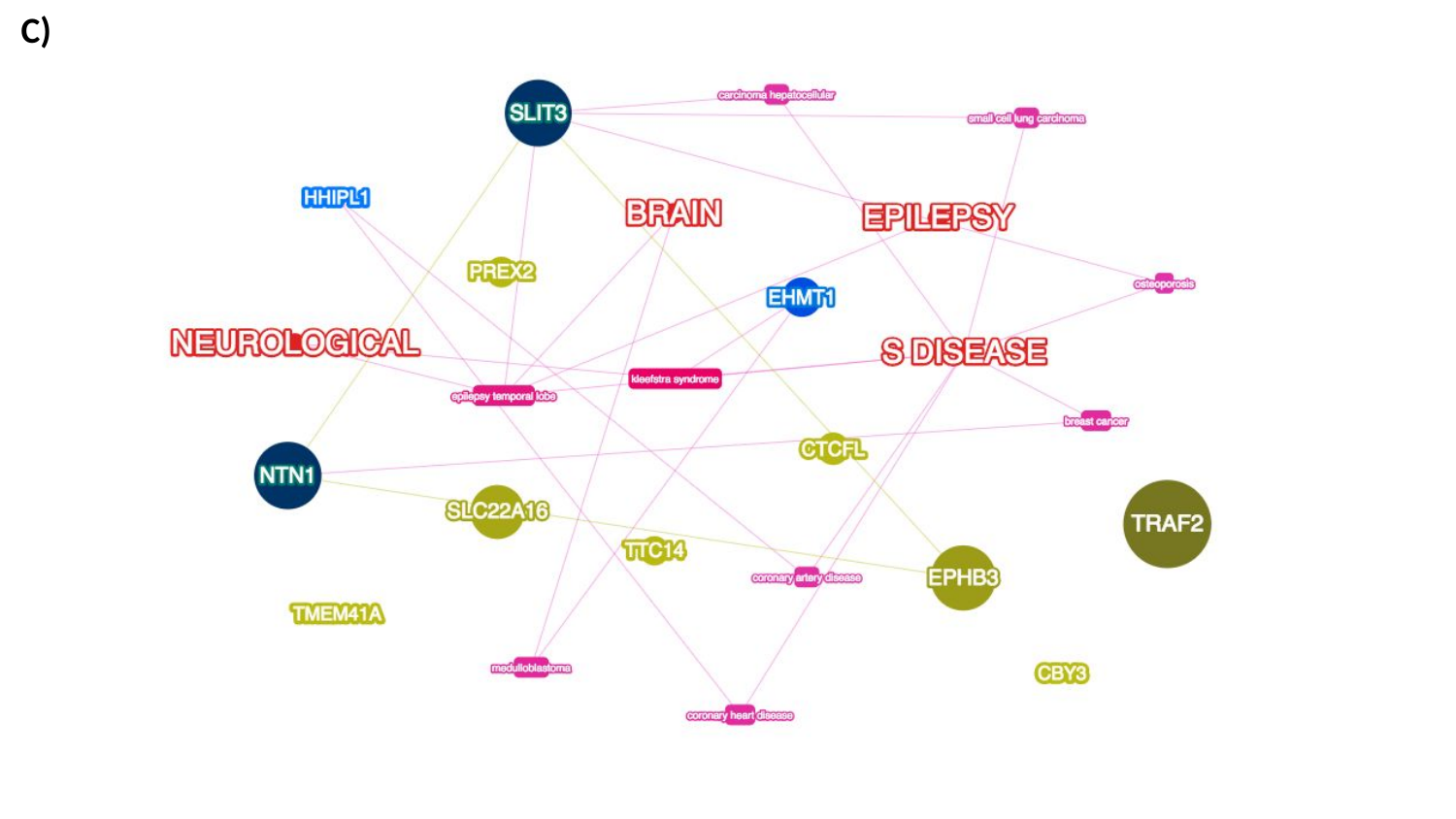

C)

## Slide 5
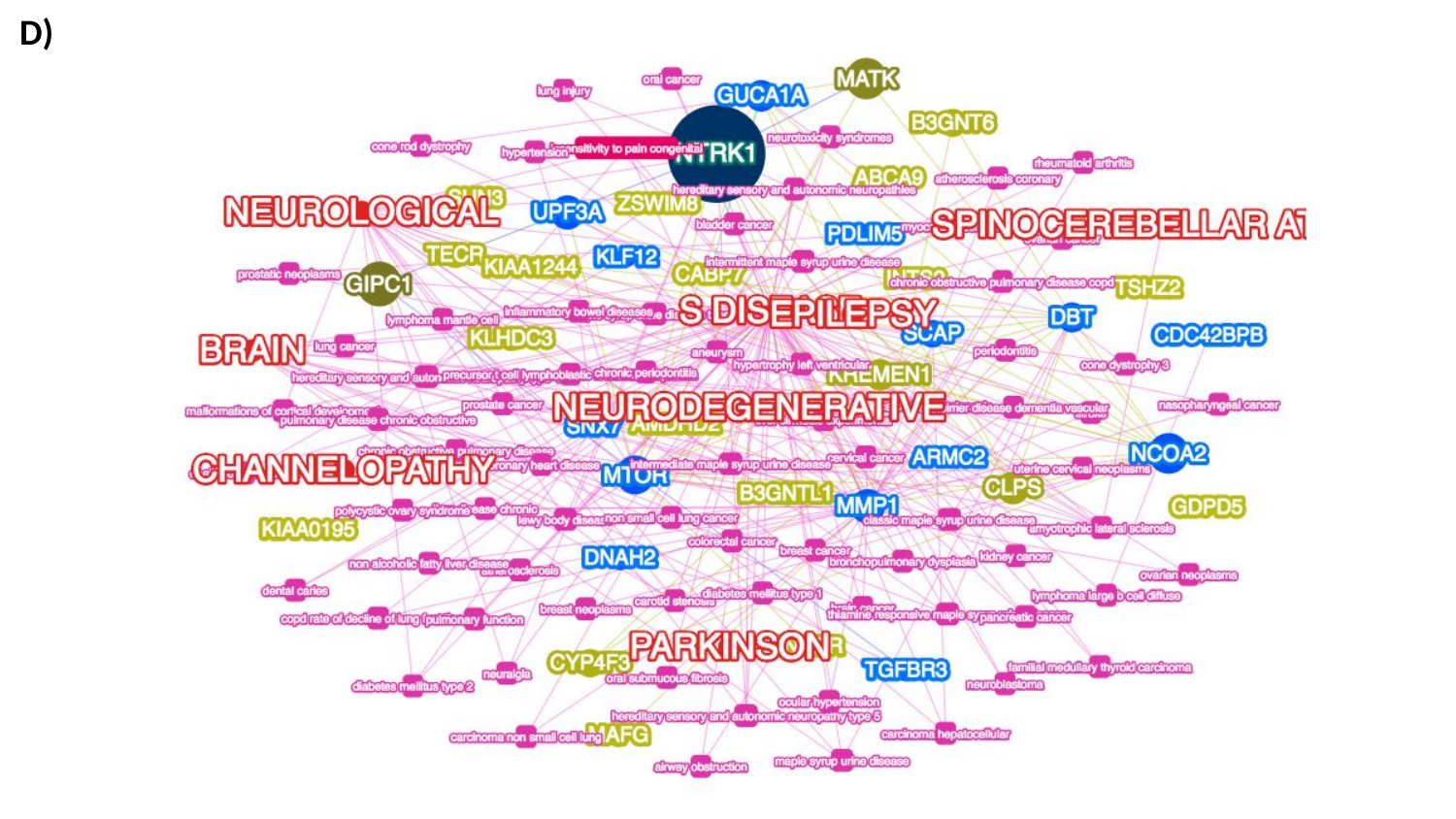

D)

## Slide 6
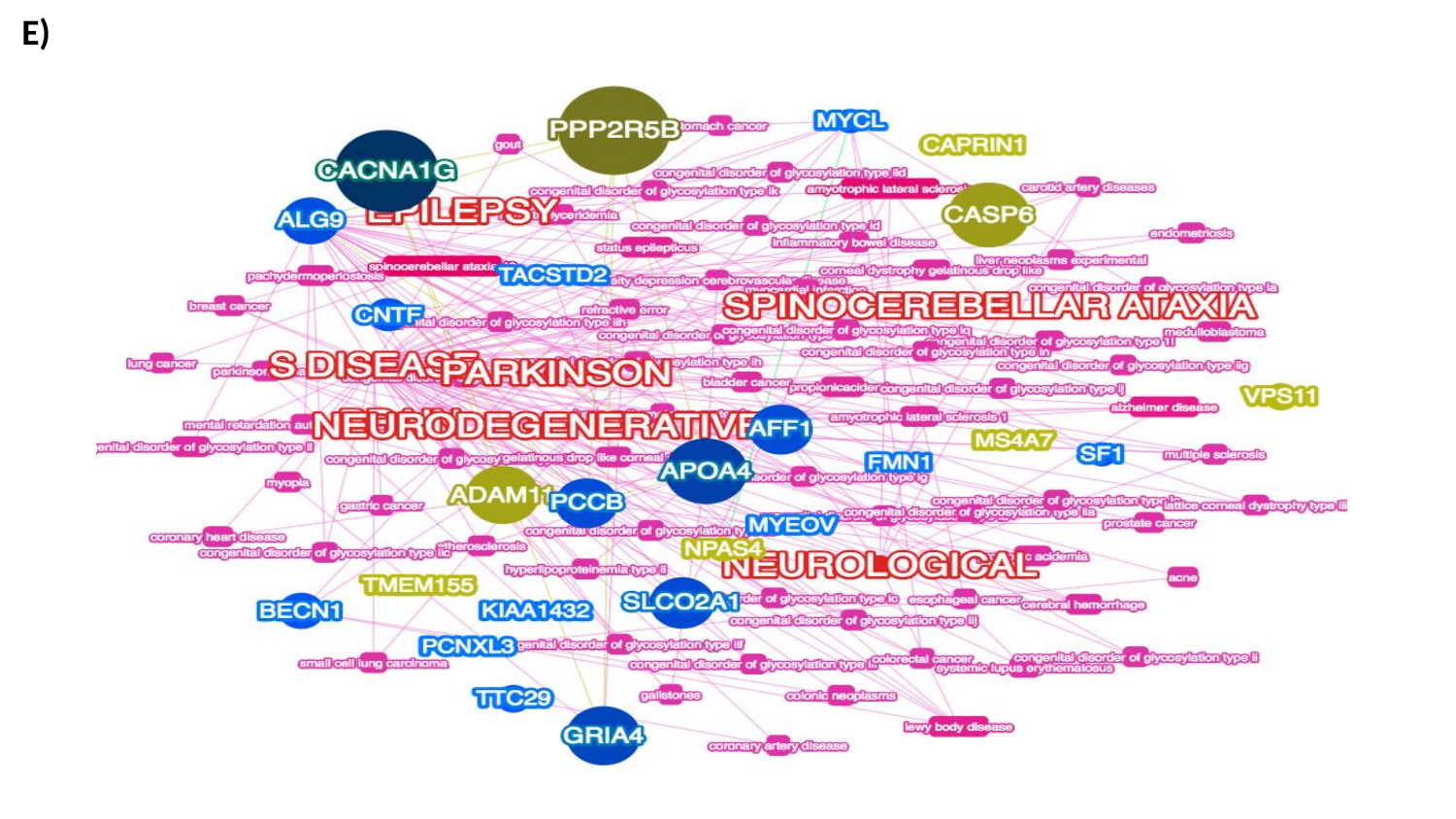

E)
