# Supplementary material for: Whole genome sequencing and rare variant analysis in essential tremor families
Source: PLoS One. 2019 Aug 12;14(8):e0220512. doi: 10.1371/journal.pone.0220512 (PMC6690583; doi:10.1371/journal.pone.0220512)

Pedigree for family with *CACNA1G* variant (c.3635G>A (NM_018896.4), p.(Arg1212Gln)), ‘+’ symbol indicates subjects were whole exome sequenced.

Pedigree for family with *CACNA1G* variant (c.1879G>A (NM_018896.4), p.(Gly627Arg)).


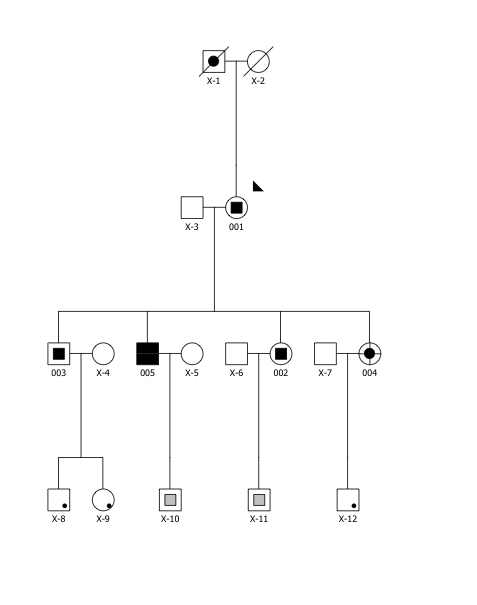

Supplement: S3 Fig — (DOCX) [file pone.0220512.s003.docx]
