# Supplementary material for: Whole genome sequencing and rare variant analysis in essential tremor families
Source: PLoS One. 2019 Aug 12;14(8):e0220512. doi: 10.1371/journal.pone.0220512 (PMC6690583; doi:10.1371/journal.pone.0220512)
Supplement: S1 Table — Variants identified in families A, B and F in WGS and WES datasets. (DOCX) [file pone.0220512.s004.docx]

**S1 Table Variants identified in Families A, B and F in WGS and WES datasets**

| **Gene** | **Chr** | **Position** | **Ref** | **Alt** | **Family** | **Called variant in WGS and WES datasets** | **Genotype Quality (GQ)**  **WES dataset per family member** | **Allelic Depths (AD) WES dataset per family member** | **Genotype Quality (GQ)**  **WES dataset per family member** | **Allelic Depths (AD) WGS dataset per family member** |
| --- | --- | --- | --- | --- | --- | --- | --- | --- | --- | --- |
| HOPX | 4 | 57514946 | G | A | A | A_G | 99* | 87,58 | 99; 99; 99; ?; 99 | 10,24; 18,17; 22,16; ?,?; 16,22 |
| EBF2 | 8 | 25902635 | C | T | A | C_T | 99* | 18,19 | 99; 99; 9; ?; 99 | 19,19; 15,16; 14,16; ?,?; 9,13 |
| ARFGEF1 | 8 | 68128883 | C | T | A | C_T | 99* | 42,47 | 99; 99; 99; ?; 99 | 16,15; 14,23, 13,18; ?,?; 20,18 |
| ZNF408 | 11 | 46723055 | - | TT | A | -/TT | 99* | 21,26 | 99; 99; 99; ?; 99 | 17,15; 19,20, 14,17; ?,?; 12,13 |
| USP22 | 17 | 20914484 | C | T | A | C_T | 99* | 14,8 | 99; 99; 99; ?; 99 | 16,18; 21,18; 14,18; ?,?; 19,11 |
| PHOSPHO1 | 17 | 47302438 | C | A | A | C_A | 99* | 17,20 | 99; 99; 99; ?; 99 | 19,17; 25,21; 16,17; ?,?; 25,12 |
| PSD4 | 2 | 113940482 | C | T | B | C_T | 99; 99; 67; 99 | 64,65; 72,85; ?,?; 70,64 | 99; 99; ?; 99; 99 | 19,19; 24,21; ?,?; 16,17; 16,16 |
| CAPN10 | 2 | 241536279 | C | T | B | C_T | 99; 96; 78; 99 | 55,75; ?,?; ?,?; 51,63 | 99; 99; ?; 99; 99 | 15,28; 13,21; ?,?; 9,14; 18,29 |
| LPCAT1 | 5 | 1477557 | G | A | B | G_A | 99; 99; 81; 99 | 28,23; 50,33; ?,?; 36,18 | 99; 99; ?; 99; 99 | 20,12; 15,16; ?,?; 9,9; 14,18 |
| ARHGEF28 | 5 | 73207339 | C | G | B | C_G | 99; 99; 99; 99 | 60,37; 55,51; ?,?; 62,76 | 99; 99; ?; 99; 99 | 20,20; 22,19; ?,?; 12,10; 19,24 |
| LOXL2 | 8 | 23177415 | C | G | B | C_G | 99; 99; 86; 99 | 23,18; 25,28; ?,?; 21,23 | 99; 99; ?; 99; 99 | 27,15; 25,21; ?,?; 10,14; 20,10 |
| B4GALNT4 | 11 | 379948 | C | T | B | C_T | 99; 99; 99; 99 | 84,83; 105,97; ?,?; 111,103 | 99; 99; ?; 99; 99 | 19,19; 18,18; ?,?; 14,10; 15,17 |
| KCNH3 | 12 | 49943258 | G | A | B | G_A | 99; 99; 99; 99 | 44,48; 51,38; ?,?; 62,67 | 99; 99; ?; 99; 99 | 24,20; 17,13; ?,?; 15,17; 18,24 |
| KRT73 | 12 | 53008439 | G | A | B | G_A | 99; 99; 60; 99 | 12,10; 12,18; ?,?; 13,14 | 99; 99; ?; 99; 99 | 17,17; 17,20; ?,?; 10,16; 20,25 |
| KIF5A | 12 | 57975211 | G | A | B | G_A | 99; 99; 96; 99 | 79,65; 66,70; ?,?; 79,80 | 99; 99; ?; 99; 99 | 23,13; 18,14; ?,?; 18,7; 27,15 |
| NDRG4 | 16 | 58537777 | A | G | B | A_G | 99; 99; 70; 99 | 41,39; 44,29; ?,?; 36,59 | 99; 99; ?; 99; 99 | 18,18; 22,14; ?,?; 16,12; 22,25 |
| MTOR | 1 | 11307911 | A | T | F | A_T | 99; 99; 99; 99 | 45,36; 35,41; 35,45; 80,53 | 99; 99; ?; 99; 99 | 13,20; 10,15; 10,17; 21,22 |
| TGFBR3 | 1 | 92161298 | T | A | F | T_A | 99; 99; 99; 99 | 53,37; 32,49; 51,32; 69,60 | 99; 99; 99; 99 | 15,19; 12,15; 16,16; 25,20 |
| SCAP | 3 | 47458897 | C | A | F | C_A | 99; 99; 99; 99 | 12,18; 20,21; 19,21; 34,35 | 99; 99; 99; 99 | 9,12; 11,9; 11,11; 15,13 |
| PDLIM5 | 4 | 95496916 | G | A | F | G_A | 99; 99; 99; 99 | 62,63; 75,76; 57,61; 80,76 | 99; 99; 99; 99 | 14,22; 13,23; 12,15; 23,18 |
| GUCA1A | 6 | 42146612 | A | G | F | A_G | 99; 99; 99; 99 | 47,53; 44,49; 42,39; 61,61 | 99; 99; 99; 99 | 16,20; 13,12; 16,9; 23,28 |
| KLHDC3 | 6 | 42986134 | C | A | F | C_A | 99; 99; 99; 99 | 79,58; 63,71; 60,59; 103,83 | 99; 99; 99; 99 | 15,23; 12,15; 10,20; 20,18 |
| SUN3 | 7 | 48033927 | C | T | F | C_T | 99; 99; 99; 99 | 17,15; 61,31; 23,17; 20,23 | 99; 99; 99; 99 | 15,13; 14,12; 17,20; 25,10 |
| ZSWIM8 | 10 | 75556529 | C | T | F | C_T | 99; 99; 99; 99 | 42,41; 34,21; 36,43; 76,52 | 99; 99; 99; 99 | 16,17; 20,16; 19,11; 26,14 |
| GDPD5 | 11 | 75167849 | AT | - | F | AT/- | 99; 99; 99; 99 | 17,17; 24,21; 23,16; 26,29 | 99; 99; 99; 99 | 21,18; 14,15; 15,13; 27,22 |
| B3GNT6 | 11 | 76750976 | T | A | F | T_A | 99; 99; 99; 99 | 28,28; 32,31; 39,32; 65,73 | 99; 99; 99; 99 | 10,24; 12,14; 12,12; 22,28 |
| MMP1 | 11 | 102668089 | G | T | F | G_T | 99; 99; 99; 99 | 36,36; 45,46; 44,62; 61,58 | 99; 99; 99; 99 | 22,22; 18,14; 13,20; 16,12 |
| KLF12 | 13 | 74420487 | G | A | F | G_A | 99; 99; 99; 99 | 9,8; 10,9; 6,7; 10,6 | 99; 99; 99; 99 | 14,20; 13,11; 16,13; 19,18 |
| UPF3A | 13 | 115047277 | G | T | F | G_T | 99; 99; 99; 99 | 28,21; 13,10; 14,17; 27,28 | 99; 99; 99; 99 | 13,17; 17,11; 12,10; 14,15 |
| AMDHD2 | 16 | 2578297 | C | T | F | C_T | 99; 99; 99; 99 | 20,26; 23,25; 37,29; 36,48 | 99; 99; 99; 99 | 10,16; 13,19; 16,15; 22,14 |
| DNAH2 | 17 | 7722271 | G | A | F | G_A | 99; 99; 99; 99 | 11,14; 13,18; 13,17; 14,17 | 99; 99; 99; 99 | 19,16; 18,18; 12,19; 18,23 |
| INTS2 | 17 | 60003873 | C | T | F | C_T | 99; 99; 99; 99 | 33,38; 33,41; 46,42; 60,50 | 99; 99; 99; 99 | 21,13; 15,18; 8,9; 16,31 |
| ABCA9 | 17 | 67039819 | G | T | F | G_T | 99; 99; 99; 99 | 42,25; 48,32; 34,45; 87,47 | 99; 99; 99; 99 | 13,19; 12,16; 17,9; 16,25 |
| B3GNTL1 | 17 | 80918994 | C | T | F | C_T | 99; 99; 99; 99 | 14,11; 13,10; 8,12; 12,18 | 99; 99; 99; 99 | 15,18; 21,16; 9,13; 16,17 |
| TECR | 19 | 14674625 | G | A | F | G_A | 99; 99; 99; 99 | 32,13; 33,16; 29,21; 34,42 | 99; 99; 99; 99 | 17,18; 20,16; 13,16; 26,23 |

*Variant only called in one family member from Family A; ‘?’ indicates missing data
