# Supplementary material for: Whole genome sequencing and rare variant analysis in essential tremor families
Source: PLoS One. 2019 Aug 12;14(8):e0220512. doi: 10.1371/journal.pone.0220512 (PMC6690583; doi:10.1371/journal.pone.0220512)
Supplement: S2 Table — Variants called in the WGS dataset but not the WES dataset in families A, B and F. (DOCX) [file pone.0220512.s005.docx]

**S2 Table Variants called in the WGS dataset but not the WES dataset in families A, B and F**

| **Gene** | **Chr** | **Position** | **Gene location** | **Ref** | **Alt** | **UCSC Genome Browser Mappability score -Uniqueness** | **UCSC Genome Browser Mappability score -Alignability** | **UCSC Genome Browser**  **Mean**  **GC %**  **(GC5base)** | **Family** | **Reason for no call*** |
| --- | --- | --- | --- | --- | --- | --- | --- | --- | --- | --- |
| AHNAK | 11 | 62283386 | 3’UTR | A | C | 1 | 1 | 19.685 (+/-27.8389) | A | A,B |
| AKAP3 | 12 | 4735970 | Exon 5 | A | - | 1 | 1 | 59.8425 (+/-0) | A | A,B |
| AMBRA1 | 11 | 46450229 | Intron | G | A | 0.33 | 0.055 | 59.8425 (+/-0) | A | A,B,D |
| CCL15 | 17 | 34325326 | Exon 3 | G | T | 1 | 1 | 59.8425 (+/-0.0045) | A | A,B |
| FLG | 1 | 152283742 | Exon 3 | G | T | 0 | 0.1 | 79.8425 (+/-0) | A | A,B,C |
| KARS | 16 | 75663435 | Exon 13 | G | A | 1 | 1 | 79.5276 (+/-0) | A | A,B,C |
| POTEI | 2 | 131221045 | Exon 15 | C | T | 0.25 | 0.125 | 79.5276 (+/-0) | A | A,B,C |
| ST18 | 8 | 53321917 | Exon 2 | C | T | 1 | 1 | 39.3701 (+/-0) | A | A,B |
| YIF1A | 11 | 66053439 | Intron | C | T | 1 | 1 | 59.8425 (+/-0) | A | A,B,D |
| BRDT | 1 | 92445126 | Exon 8 (intron/exon boundary) | G | A | 1 | 1 | 39.3701(+/-0.01) | B | A,B |
| CBWD5 | 9 | 70483186 | intron | A | G | 0.25 | 0.2 | 39.7683(+/-28.3956) | B | A,B |
| CIZ1 | 9 | 130953056 | Exon 2 | - | CTGCTGGAG  CTGCTGCTGCTGTAA | 1 | 1 | 59.685 (+/-14.1995) | B | A,B |
| DPYD | 1 | 98205947 | Intron | C | T | 1 | 1 | 27.5591 (+/-17.6068) | B | A,B,D |
| FAM129B | 9 | 130272601 | Exon 9 | G | C | 1 | 1 | 69.685 (+/-13.9194) | B | A,B,C |
| POTEI | 2 | 131221215 | Exon 15 | T | A | 1 | 0.333 | 79.5276 (+/-0) | B | A,B,C |
| ADAM28 | 8 | 24193085 | Exon 14 | G | A | 1 | 1 | 59.8425 (+/-0) | B | A,B |
| ARMC2 | 6 | 109200145 | Intron | CATCCAC  CCAGACACCCATT | - | 1 | 0.666 | 47.5591 (+/-22.8251) | F | A,B,D |
| CABP7 | 22 | 30116623 | Intron | G | A | 1 | 1 | 79.5276 (+/-0) | F | A,B,C,D |
| CDC42BPB | 14 | 103404716 | Exon 35 | C | T | 1 | 1 | 100(+/-0) | F | A,B,C |
| CLPS | 6 | 35765011 | Exon 1 | G | A | 1 | 1 | 69.685 (+/-13.9194) | F | A,B,C |
| CYP4F3 | 19 | 15770059 | Intron | C | A | 1 | 0.16667 | 79.5276(+/-0) | F | A,B,C,D |
| DBT | 1 | 100679506 | Intron | A | - | 1 | 0.3333 | 19.685 (+/-27.8389) | F | A,B |
| GIPC1 | 19 | 14593508 | Exon 4 | G | A | 1 | 1 | 39.3701(+/-0) | F | A,B |
| KIAA0195 | 17 | 73485444 | Exon 8 | G | A | 1 | 1 | 59.8425 (+/-0) | F | A,B |
| KIAA1244 | 6 | 138582682 | Exon 11 | C | T | 1 | 1 | 79.5276(+/-0) | F | A,B,C |
| KREMEN1 | 22 | 29545589 | Intron | G | A | 1 | 1 | 100(+/-0) | F | A,B,C |
| MAFG | 17 | 79885565 | Exon 1 (5’UTR) | C | G | 1 | 1 | 79.5276(+/-0) | F | A,B,C |
| MATK | 19 | 3786302 | Exon 1 (5’UTR) | A | G | 1 | 1 | 19.685 (+/-0) | F | A,B |
| NCOA2 | 8 | 71036930 | Exon 20 | C | T | 1 | 1 | 39.3701(+/-0) | F | A,B |
| SNX7 | 1 | 99127352 | Non-coding exon 1 | G | A | 1 | 1 | 79.5276(+/-0) | F | A,B,C |
| TSHZ2 | 20 | 52109752 | Exon 3 | A | G | 1 | 1 | 19.685 (+/-0) | F | A,B |
| NTRK1 | 1 | 156814612 | Intron | T | C | 1 | 1 | 61.7074 (+/-16.231) | F | A, B, D |

*A-poor depth of coverage filtered out, B-poor genotype quality filtered out, C-variant located in GC rich region, D-intronic variant not captured in WES
